# Supplementary material for: Rank-dependent control of tuft and BEST4 cell development in the intestine
Source: Nat Commun. 2026 May 19;17:6609. doi: 10.1038/s41467-026-73293-9 (PMC13381698; doi:10.1038/s41467-026-73293-9)
Supplement: Supplementary file 1 — Supplementary Information [file 41467_2026_73293_MOESM1_ESM.pdf]

SUPPLEMENTARY FIGURES

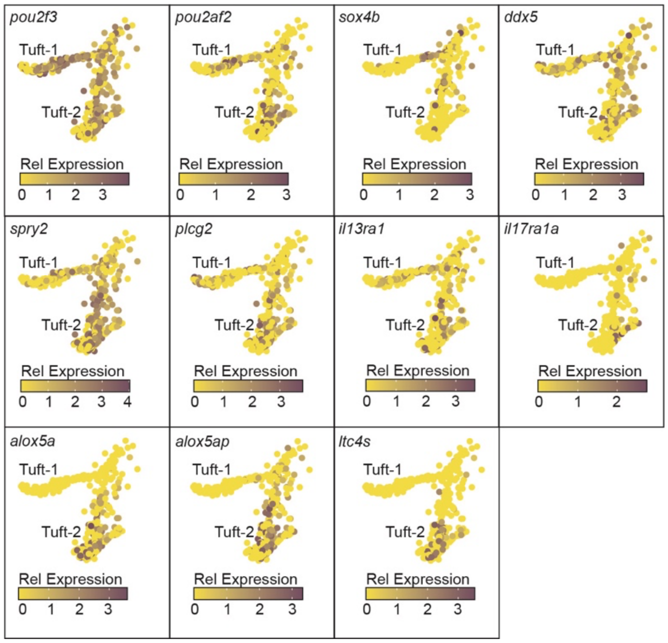

Figure S1. Expression of classical tuft cell markers (panels from *pou2f3* to *plcg2*) and immune regulatory markers (panels from *il13ra1* to *ltc4s*) in zebrafish tuft cells.

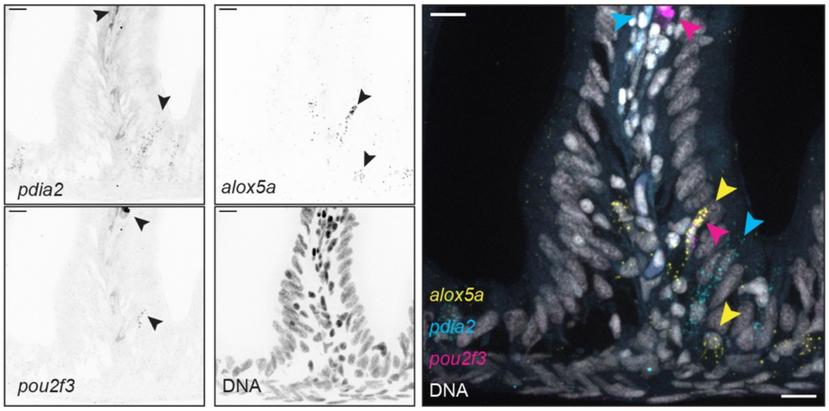

Figure S2. Fluorescence *in situ* hybridization showing the expression of the indicated genes in grayscale for each gene, and pseudo colored in the merged image. In each image, scalebars are twenty micrometers, and arrowheads point to cells that express the gene of interest.

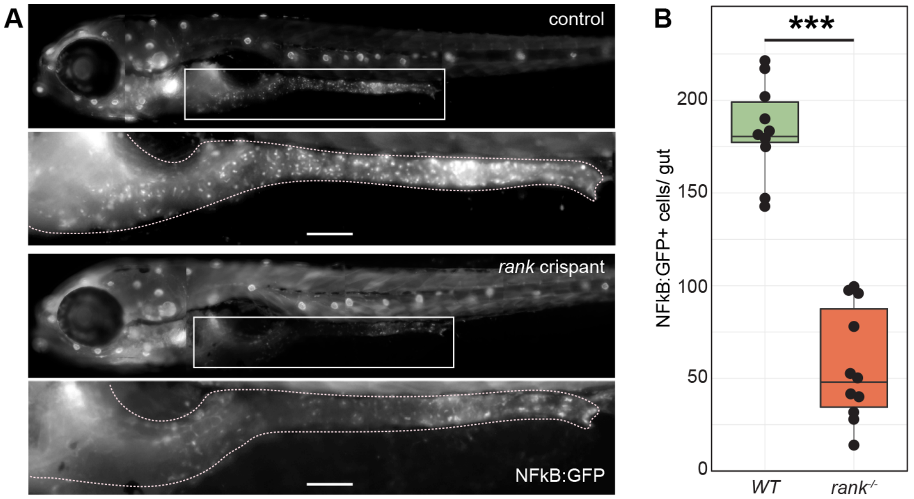

Figure S3. Visualization (A) and quantification (B) of GFP expression in control (n = 10) or *rank* crispant (n = 11) NF-kB:GFP 6 dpf larvae. Box plots show the median (centre line), 25th and 75th percentiles (box limits), and most extreme non-outlier values (whiskers). Significance between the two genotypes was tested using a Mann-Whitney U test. Asterisks indicate a P value below 0.001 (p = 7.46E-09).

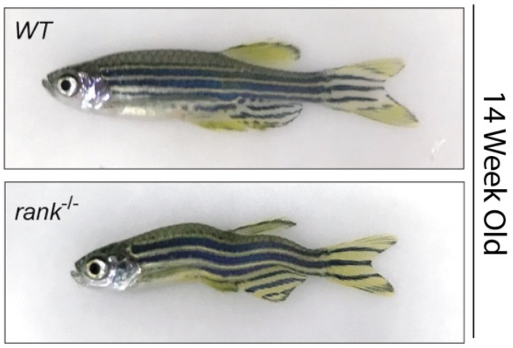

Figure S4. Spinal curvature defects in fourteen-week-old *rank* mutant fish relative to wildtype (WT) controls.

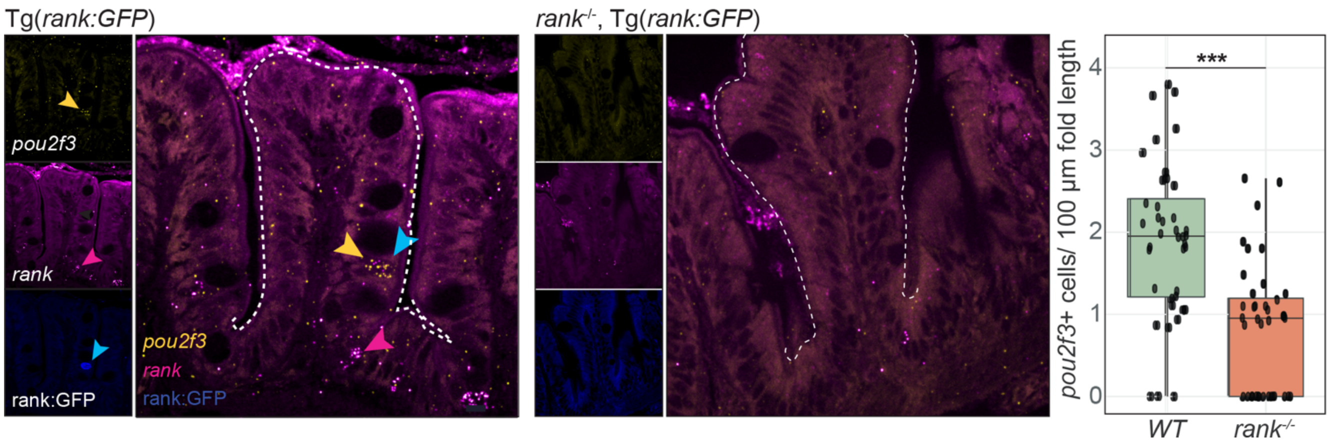

Figure S5: Visualization of the expression of *pou2f3*, *rank*, and GFP in wildtype (Tg(*rank:GFP*)) and RANK-deficient (rank-/-, Tg(*rank:GFP*)) adult zebrafish intestines. Arrowheads indicate cells expressing the gene of interest, and a single fold is indicated with a dashed line in both merged images. The boxplot shows the percentage of *pou2f3*+ cells normalized to fold length for the indicated genotypes. Data were gathered from five fish, multiple regions of interest were analyzed per fish and all measurements are displayed. Box plots show the median (centre line), 25th and 75th percentiles (box limits), and most extreme non-outlier values (whiskers). Significance between the two genotypes was tested using a Mann-Whitney U test. Asterisks indicate a P value below 0.001 ((p = 1.9E-06).

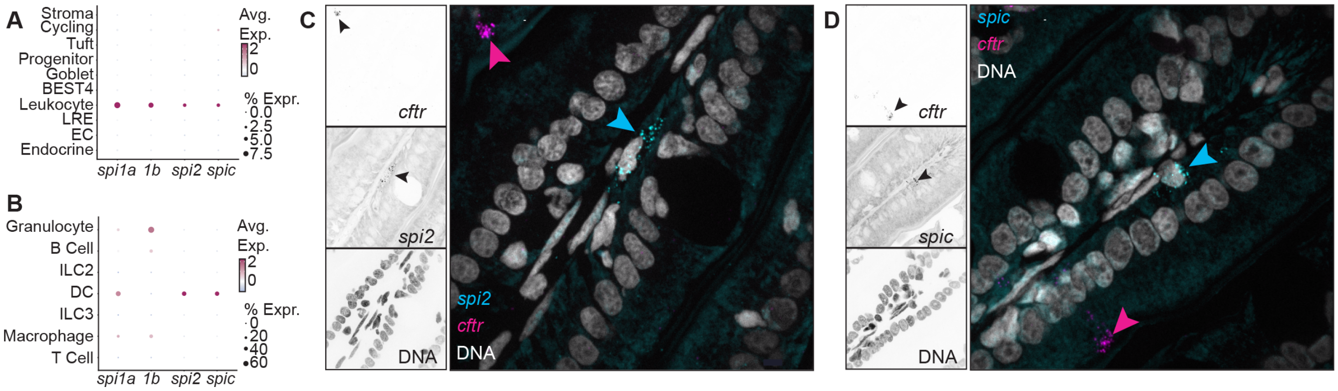

Figure S6. A-B: Expression patterns of zebrafish *spi* family members in the zebrafish guts (A) and leukocyte cell types (B). **C-D:** Visualization of *cftr* co-expression with *spi2* (C) and *spic* (D) in adult zebrafish intestines. Whereas *cftr* marks BEST4 cells, *spi2* and *spic* mark vasculature-associated dendritic cells.

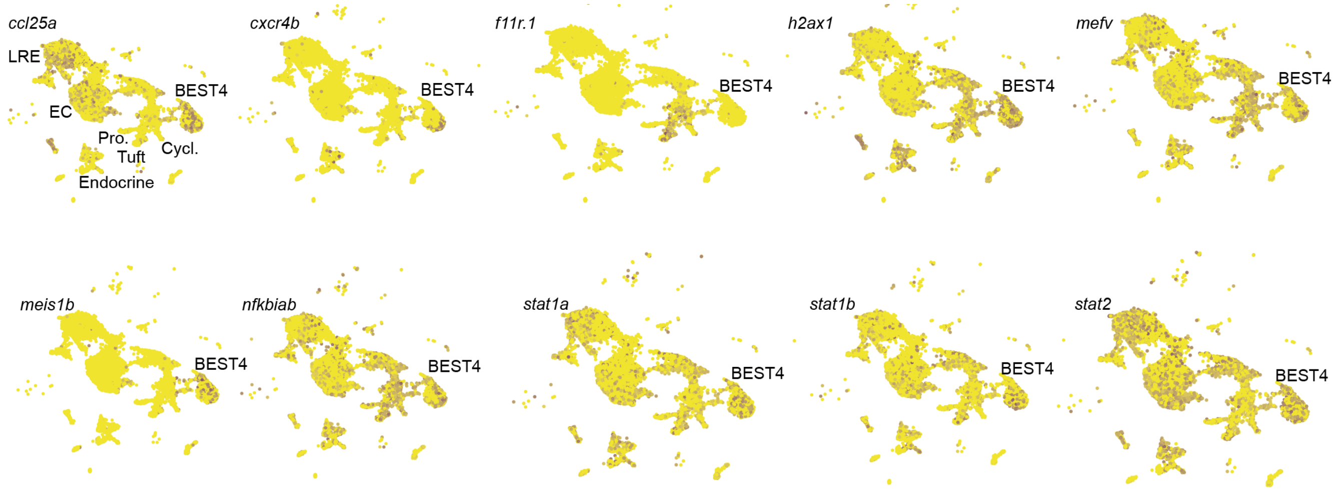

Figure S7. A series of feature plots showing expression of multiple immune regulatory genes in adult intestinal BEST4 cells.

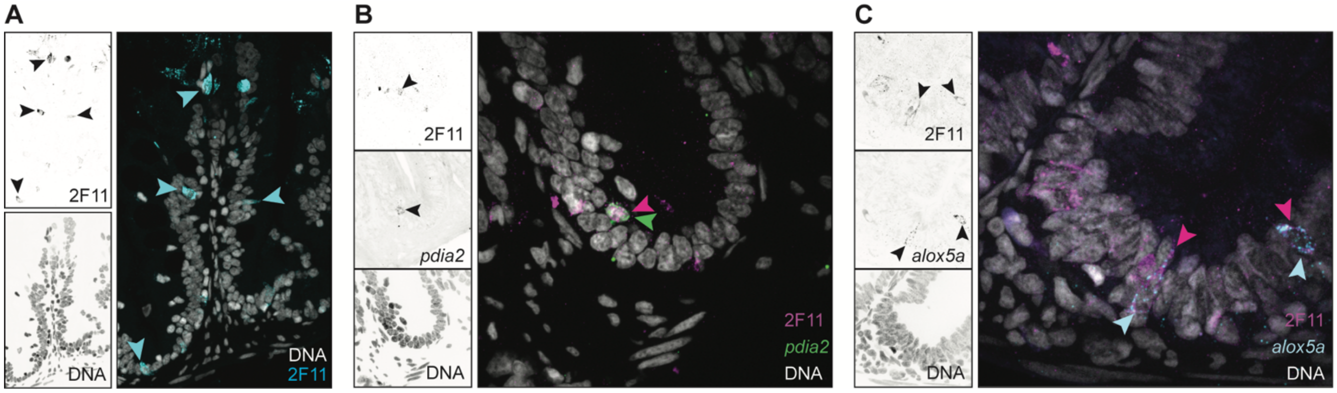

Figure S8. A: Visualization of 2F11+ cells (marked with arrowheads) in the intestinal epithelium of an adult zebrafish. **B-C:** Dual staining of intestines for 2F11 (B-C) and *pdia2* (B) or *alox5a* (C). In the false-colored images, DNA is labeled in grey, 2F11 is labeled in magenta C, *pdia2* is labeled green B, and *alox5a* is labeled cyan C. In each case, the tuft cell markers overlap with the 2F11 antibody that labels epithelial secretory cells.
