# Supplementary material for: Rank-dependent control of tuft and BEST4 cell development in the intestine
Source: Nat Commun. 2026 May 19;17:6609. doi: 10.1038/s41467-026-73293-9 (PMC13381698; doi:10.1038/s41467-026-73293-9)
Supplement: Supplementary file 3 — Description of Additional Supplementary Files [file 41467_2026_73293_MOESM3_ESM.pdf]

Description of Additional Supplementary Files

File Name: Supplementary Data 1
Description: Relative expression (represented as log2 Fold Change) of immune related genes in rank mutants relative to wildtype siblings.
