# Supplementary material for: Rank-dependent control of tuft and BEST4 cell development in the intestine
Source: Nat Commun. 2026 May 19;17:6609. doi: 10.1038/s41467-026-73293-9 (PMC13381698; doi:10.1038/s41467-026-73293-9)
Supplement: Supplementary file 5 — Reporting Summary [file 41467_2026_73293_MOESM5_ESM.pdf]

nature portfolio

Corresponding author(s): Edan Foley

Last updated by author(s): April 30, 2026

Reporting Summary

Nature Portfolio wishes to improve the reproducibility of the work that we publish. This form provides structure for consistency and transparency in reporting. For further information on Nature Portfolio policies, see our Editorial Policies and the Editorial Policy Checklist.

Statistics

For all statistical analyses, confirm that the following items are present in the figure legend, table legend, main text, or Methods section.

n/a	Confirmed	
☐	☒	The exact sample size (*n*) for each experimental group/condition, given as a discrete number and unit of measurement
☐	☒	A statement on whether measurements were taken from distinct samples or whether the same sample was measured repeatedly
☐	☒	The statistical test(s) used AND whether they are one- or two-sided<br>*Only common tests should be described solely by name; describe more complex techniques in the Methods section.*
☒	☐	A description of all covariates tested
☒	☐	A description of any assumptions or corrections, such as tests of normality and adjustment for multiple comparisons
☐	☒	A full description of the statistical parameters including central tendency (e.g. means) or other basic estimates (e.g. regression coefficient) AND variation (e.g. standard deviation) or associated estimates of uncertainty (e.g. confidence intervals)
☐	☒	For null hypothesis testing, the test statistic (e.g. *F*, *t*, *r*) with confidence intervals, effect sizes, degrees of freedom and *P* value noted<br>*Give P values as exact values whenever suitable.*
☒	☐	For Bayesian analysis, information on the choice of priors and Markov chain Monte Carlo settings
☒	☐	For hierarchical and complex designs, identification of the appropriate level for tests and full reporting of outcomes
☒	☐	Estimates of effect sizes (e.g. Cohen's *d*, Pearson's *r*), indicating how they were calculated

Our web collection on statistics for biologists contains articles on many of the points above.

Software and code

Policy information about availability of computer code

Data collection	No software was used.
Data analysis	nf-core RNA-seq pipeline FASTQC (version 0.11.9); TrimGalore! (version 0.6.7); STAR (version 2.7.10a) Salmon (version 1.5.2); DESeq2 (version 1.38.3); DAVID gene ontology analysis; R (version 2024.12.0+467); Seurat (version 5.4.0); ggplot2 (version 4.0.1)

For manuscripts utilizing custom algorithms or software that are central to the research but not yet described in published literature, software must be made available to editors and reviewers. We strongly encourage code deposition in a community repository (e.g. GitHub). See the Nature Portfolio guidelines for submitting code & software for further information.

Data

Policy information about availability of data

All manuscripts must include a data availability statement. This statement should provide the following information, where applicable:

- Accession codes, unique identifiers, or web links for publicly available datasets
- A description of any restrictions on data availability
- For clinical datasets or third party data, please ensure that the statement adheres to our policy

Bulk RNA-seq data files have been deposited to the NCBI GEO database (NCBI GEO(GSE303709)). We have instructed NCBI to make the data publicly available

Research involving human participants, their data, or biological material

Policy information about studies with human participants or human data. See also policy information about sex, gender (identity/presentation), and sexual orientation and race, ethnicity and racism.

Reporting on sex and gender	N/A
Reporting on race, ethnicity, or other socially relevant groupings	N/A
Population characteristics	N/A
Recruitment	N/A
Ethics oversight	N/A

Note that full information on the approval of the study protocol must also be provided in the manuscript.

Field-specific reporting

Please select the one below that is the best fit for your research. If you are not sure, read the appropriate sections before making your selection.

[x] Life sciences [ ] Behavioural & social sciences [ ] Ecological, evolutionary & environmental sciences

For a reference copy of the document with all sections, see nature.com/documents/nr-reporting-summary-flat.pdf

Life sciences study design

All studies must disclose on these points even when the disclosure is negative.

Sample size	Sample size was not predetermined using formal power calculations but was based on established practices in the field and prior experience with this experimental system. A sample size of five biological replicates per group was sufficient to detect robust and statistically significant differences between conditions. No samples were excluded from analysis.
Data exclusions	no data were excluded from analysis
Replication	All experiments were repeated several times to confirm replicability.
Randomization	All experiments were independently repeated to confirm reproducibility, with consistent results obtained across replicates.
Blinding	For quantitative analyses, researchers were blinded to sample identity during data acquisition and analysis.

Reporting for specific materials, systems and methods

We require information from authors about some types of materials, experimental systems and methods used in many studies. Here, indicate whether each material, system or method listed is relevant to your study. If you are not sure if a list item applies to your research, read the appropriate section before selecting a response.

Materials & experimental systems

n/a	Involved in the study	
[ ]	[x]	Antibodies
[x]	[ ]	Eukaryotic cell lines
[x]	[ ]	Palaeontology and archaeology
[ ]	[x]	Animals and other organisms
[x]	[ ]	Clinical data
[x]	[ ]	Dual use research of concern
[x]	[ ]	Plants

Methods

n/a	Involved in the study	
[x]	[ ]	ChIP-seq
[x]	[ ]	Flow cytometry
[x]	[ ]	MRI-based neuroimaging

Antibodies

Antibodies used	Chicken polyclonal anti-GFP (ThermoFisher Scientific Cat# PA1-9533); 2F11 (abcam Cat# ab71286); Goat anti-chicken IgY (H+L) secondary antibody, Alexa Fluor™ 488 (ThermoFisher Scientific Cat# A-11039); Goat anti-Rabbit IgG (H+L) Cross-Adsorbed Secondary Antibody, Alexa Fluor™ 568 (ThermoFisher Scientific Cat# A-11011)
Validation	Chicken polyclonal anti-GFP validation: https://www.thermofisher.com/antibody/primary/query/chicken%20GFP

nature portfolio | reporting summary April 2023
2F11 validation: PMID: 15689380 and manufacturer https://www.abcam.com/en-us/products/primary-antibodies/zebrafish-gutsecretory-cell-epitopes-antibody-fis-2f11-2-ab71286

Animals and other research organisms

Policy information about studies involving animals; ARRIVE guidelines recommended for reporting animal research, and Sex and Gender in Research

Laboratory animals	Danio rerio (zebrafish). Strains: Wild type TL, Tg(rank:GFP), and Tg(NFkB:GFP); rank mutant.
Wild animals	No No wild animals were used
Reporting on sex	Larvae are hermaphrodites. For experiments with adults equal numbers of of males and females were in in each control and experimental group.
Field-collected samples	No field samples were collected
Ethics oversight	All zebrafish experiments were approved by by the University of of Alberta's Animal Care & Use Committees of of Biosciences and Health Sciences (AUP#3032), operating under the guidelines of of the Canadian Council of of Animal Care

Note that full information on the approval of the study protocol must also be provided in the manuscript.

Plants

Seed stocks	N/A
Novel plant genotypes	N/A
Authentication	N/A
