# Supplementary material for: Rank-dependent control of tuft and BEST4 cell development in the intestine
Source: Nat Commun. 2026 May 19;17:6609. doi: 10.1038/s41467-026-73293-9 (PMC13381698; doi:10.1038/s41467-026-73293-9)
Supplement: Supplementary file 2 — Peer Review file [file 41467_2026_73293_MOESM2_ESM.pdf]

nature portfolio

Peer Review File

RANK-DEPENDENT CONTROL OF TUFT AND BEST4 CELL DEVELOPMENT IN THE INTESTINE

Corresponding Author: Professor Edan Foley

This file contains all reviewer reports in order by version, followed by all author rebuttals in order by version.

Version 0:

Reviewer comments:

Reviewer #2

(Remarks to the Author)
In the manuscript “Rank-dependent control of tuft and Best4 cell development in the intestine,” Willms and colleagues investigated how Rank regulates intestinal epithelial cell development in zebrafish. The authors demonstrated that systemic deletion of rank in zebrafish disrupts the maintenance of type 2-tuft cells and Best4 cells in the small intestine, altering the proportions of goblet cells. These findings illustrate how systemic Rank signaling impacts the development of intestinal epithelial cells and suggest potential physiological consequences of systemic rank deficiency.
However, a major limitation of this manuscript is the lack of investigation into the physiological relevance of the observed changes in epithelial cell development in rank-knockout zebrafish. Second, the data do not elucidate which downstream signaling pathways are involved in the maintenance of type-2 tuft cells and Best4+ cells. Moreover, some observations demonstrate a distinct role of Rank in the zebrafish, which is controversial in certain aspects, particularly the role of Rank in the development of Best4+ cells, which has not been observed in humans (DOI: 10.1016/j.stem.2025.02.003). However, as the authors in this manuscript only use full-body rank-knockout zebrafish, it remains unclear if the effects of Rank on Best4+ cell development are dependent on a direct role of Rank in the intestinal epithelial cells or secondarily from the effects of Rank in the other type of cells or other tissues.
RANK is well known to modulate immune cell functions, including self-tolerance in the thymus, lymph node organogenesis, and T cell activation, through signaling in thymic epithelial cells, lymphoid tissue inducer (LTi) cells, endothelial cells, and dendritic cells (doi:10.1038/16852; doi: 10.1101/gad.13.18.2412; DOI: 10.1016/j.immuni.2008.06.015). These immune-related alterations could happen in rank-deficient zebrafish, thereby likely influencing intestinal epithelial homeostasis as a secondary effect, since many previous reports demonstrated the maintenance of intestinal stem cells by immune cells. Thus, the changes observed in epithelial cell fate may result from indirect, immune-mediated effects rather than the direct effects of Rank signaling in the intestinal epithelial cells. The use of a full-body rank knockout model alone is insufficient to determine the specific role of Rank in the intestinal epithelial cells.
To directly assess the epithelial-intrinsic role of Rank signaling, the authors should use either a conditional knockout strategy using a floxed rank allele with an intestinal-specific Cre driver or investigate zebrafish-derived intestinal organoid systems. These approaches would enable a more precise dissection of the cell-autonomous effects of Rank on intestinal epithelial cell development.
The authors need to address the following questions listed below.

Major comments;
1. The authors showed that rank expression on tuft cell precursors and type-2 tuft cells among their lineage cells. The authors should display the expression of rank in the entire intestinal tissue from scRNA-seq data, including all subtypes of intestinal epithelial cells, immune cells, and mesenchymal cells localized in the intestine. An overview of the rank expression in cells localized in the intestine will help elucidate in which cell types the impairment of Rank signaling occurs, as well as which cells may affect the fate of the intestinal cell lineage. Moreover, if the Rank signaling in the intestine affects Best4 cells’ development, rank is also likely expressed on their lineage.

2. It would be better to show the expression of spib, the master regulator of tuft cells in mice and humans (DOI: 10.1126/sciimmunol.ads5818; DOI: 10.1016/j.stem.2025.02.003), and sh2d6, as a marker for tuft-2 cells observed in mice, in Fig. 1D and F (https://doi.org/10.1016/j.immuni.2022.03.001).

3. In the last sentences on page 5 and Fig. 1J, the authors mentioned that ‘We found that alox5a-positive cells preferentially accumulated at the villus base,’ however, the images in Fig. 1H show the localization of alox5a-positive cells in the apical site of villi. Usually, type-2 tuft cells localize in the tip of the villi as more differentiated tuft cells, as shown in the reference paper (https://doi.org/10.1016/j.immuni.2022.03.001). The authors need to clarify these data.

4. In Fig. 2G, the authors showed that the pou2f3+ cells (pan-tuft cell marker) are barely detectable in rank KO zebrafish, suggesting that Rank signaling is essential for the development of whole tuft cells, including both type-1 and type-2; however, in Fig. 2C-E, the number of pdia2+ cells (type1 tuft cell marker) are comparable between WT and rank KO fish, implying that Rank signaling is not essential for development of type-1 tuft cells. The authors should reconcile these conflicting data.

5. Fig. 2D-F shows that rank-deletion causes the disapperance of rank-GFP-positive cells, which presumably include both her6, her9-expressing precursor cells and differentiated tuft cells. It appears that Rank signaling is essential not only for tuft cell development but also for maintaining those precursor cells. The authors need to argue the results more precisely.

6. The authors characterized leukocytes localized in the gut in Fig. 3. As observed in the whole body Rankl and Rank knockout mice (doi:10.1038/16852; doi: 10.1101/gad.13.18.2412), RANK-RANKL signaling controls lymph node organogenesis, extramedullary hematopoiesis. Thus, the different characters and the accumulation of immune cells in the intestine of rank-KO zebrafish may derive from dysregulation of immune cell development in lymphatic tissues.
The author mentioned that rank deficiency shifts the hematopoietic compartment to a pro-inflammatory state. It would be meaningful if the authors addressed whether the intestinal tissues suffer from tissue damage, tissue inflammation, or cause intestinal barrier breakdown. Do rank KO zebrafish have higher sensitivity to any experimental colitis?
Moreover, although the author’s subtitle for this section is ‘RANK deficiency causes leukocyte accumulation in the gut,’ the number of Lcp1+ cells seems only subtly different between WT and KO zebrafish, even though there is a significant difference between samples. It would be better to change the title towards a direction that more emphasizes the proinflammatory state of infiltrated immune cells. The authors need to investigate which cell types, such as T cells, B cells, monocytes/macrophages, granulocytes, and dendritic cells, are differentially accumulated in the intestine.

7. Fig.4A shows that the cell localization of alox5a-positive and rank-positive cells in the WT. Some of the cells localize close to the muscle layer, suggesting that there is a possibility that those positive cells are not epithelial cells, but other cell types localized in the lamina propria, such as immune cells or mesenchymal cells. To show the exact localization of positive cells and determine the cell type of those positive cells, the authors should co-stain the tissue with F-actin or an epithelial cell marker such as Epcam.

8. In Fig. 5B, the authors should include ca7, the major Best4 cell marker, and spib, an important transcription factor (DOI: 10.1016/j.stem.2025.02.003).

9. What happens to the rank KO zebra fish after Vibrio cholerae challenge? Do the rank KO mice show difficulty in eliminating Vibrio cholerae and persistence of infection because of a lack of Best4+ cells as ionocytes? The authors should address the physiological outcome of the reduction of Best4+ cells in rank KO zebrafish.

10. In lines 1-7, the authors discussed the discrepancy of the role of Rank signaling in the gut on Microfold cells and BEST4 cell differentiation between the data from the authors and the other papers. The authors need to point out that their data come from full-body knockout zebrafish, and this model might not focus on the role of Rank signaling in the intestine.

Minor points;
1. The legend for Figure 6D is missing. And the numbering of the figure legends in Fig.6D-H is wrong.

2. The author should explain or argue why the goblet cell signature gene expression is not altered, even though the number of the goblet cells is different between WT and KO in Fig. 2L and 6D.

3. The author should mention which statistic was used for Figs. 4 and 5, respectively.

4. In the last line on page 18, ‘appear to’ is duplicated.

Reviewer #3

(Remarks to the Author)
I co-reviewed this manuscript with one of the reviewers who provided the listed reports. This is part of the Nature Communications initiative to facilitate training in peer review and to provide appropriate recognition for Early Career Researchers who co-review manuscripts.

Reviewer #4

(Remarks to the Author)
OVERALL IMPRESSION
This is a nice manuscript that I think will be of broad interest and of impact within the intestinal biology community, with several novel results. I think that there are some results or analyses presented that are overinterpreted in the manuscript and

should be revised. Additionally, I think that an expanded discussion that makes more clear some of the limitations of the conclusions of the manuscript is likely warranted. The Methods section currently omits several experiments or analyses.

SUMMARY OF RESULTS

Reems, McCabe, Jones, Schade, and Foley demonstrate that:
- Zebrafish have multiple types of tuft / tuft-like cells (alox5a and pdia2) that are found in both adults and larvae and have different distributions along the villus axis. These types express different genes, with the alox5a population expressing inflammatory genes linked to type two inflammation.
- RANK mutants exhibit gross morphological defects, including a shorter body length, shorter intestine, and exhibit scoliosis as they age.
- Adult RANK mutants have a complete loss of tuft / tuft-like cells, while larval RANK mutants have a severe reduction, though still maintain a small number of RANK:GFP+, pou2f3+ cells.
- RANK mutants result in increased expression of immune genes in bulk RNAseq, and show modestly increased infiltration of leukocytes in the intestine.
- RANK mutants have reduced alox5a tuft cells through the intestine and reduced pdia2 tuft cells in the anterior intestine.
- Adult zebrafish have BEST4 cells in all regions of the intestine, though they are enriched in the mid intestine, and along the entire length of the villus axis.
- cftr-positive cells increase after infection with Vibrio cholerae.
- RANK mutant intestines have reduced BEST4 cells in multiple regions of the intestine.
- RANK mutant intestines have reduced secretory cells, increased goblet cells, altered number of EECs, and reduced BEST4 cells.

ISSUES THAT SHOULD BE ADDRESSED:

1. The authors propose that a common progenitor directly gives rise to the two types of tuft cells in zebrafish, and provide support in the form of a transcriptional trajectory. However, recent reports from humans and mice (Buissant des Amorie et al Nature Communications 2025) suggest that the two tuft cell types "represent successive stages of maturation drive by crypt-villus signaling gradients". This would be consistent with the different localization observed in zebrafish for these two subtypes, where the tuft-2 like cells are nearer the villus tips. Additionally, while rare, the authors do see some cells that co-express markers of both tuft cell types, which could be consistent with such a transition.
a. Ideally, the authors would conduct some kind of lineage tracing to resolve the differences. One possible experiment is that the authors could potentially use a transgenic marker of pdia2 tuft cells to confirm that alox5a tuft cells are not actually a further 'matured' state of these cells. Perhaps, however, there are simpler approaches that may address this issue?
b. Given that a lineage tracing experiment may be difficult in the timeframe of revisions of this manuscript, if not performed, it seems important to discuss the limitations of transcriptional trajectories, to discuss the mammalian lineage results that conflict with the proposed developmental relationships here, and to discuss any additional data that further suggests the two tuft-cell subtypes cannot be different maturation states of the same cell type (if it exists), and to suggest the need to future follow-up work.
2. The manuscript suggests that the two tuft-cell subtypes in zebrafish mirror the division of function of tuft-cell subtypes in mammalian systems. This is only supported with a handful of key genes, though. This point would be MUCH stronger if a broader comparison of their transcriptomes were performed — which seems in the authors' wheelhouse, based on their cell-type comparisons in Fig 5E-F. This would also strengthen their section in the discussion describing the relative cellular profiles between zebrafish and mammals and enable a stronger description of which features of mammalian tuft cells are best modeled within the zebrafish, which seems like a key point this manuscript would want to make if it is establishing that there are similar subtypes in zebrafish.
3. The manuscript overinterprets the necessity of RANK in some places. Tuft and BEST4 cells are reduced in number in RANK mutants, but not eliminated by any means. Thus, it seems that descriptions of 'necessity' or 'essentiality' are not accurate since these cell types are still capable of developing in the absence of RANK (e.g. subtitles "RANK is Necessary for Development of alox5a-positive Tuft Cells" and "RANK is Necessary for BEST4 Cell Development", as well as text references "requirement for RANK in the development of immune regulatory tuft cells", "confirming an essential role for RANK in the development of Anxa4-positive cells", and potentially others – the authors should do a comprehensive sweep). It would be more appropriate to suggest that RANK "plays a role" or "modifies development" or "influences development". Similarly "control" in the title might be too strong of a term for a partial effect?
4. Figure 2G-H: it's unclear why the assay presented switches from pou2f3+ cells to f-actin+ cells between the larva and adult. While it's interesting to also show the f-actin results (which I suppose suggest that tuft cells don't differentiate properly in RANK mutants?), since adults have also been stained for pou2f3 (Fig 1L, N), I think they should be quantified and displayed in place of 2H, though I would support keeping the f-actin result also in the main figure as a later panel. Currently, this gives the impression that tuft cells are completely lost in RANK mutants, which does not seem to be true, since there are several alox5a or pdia2 cells still in the animal (Figure 4).
5. Do the authors have any data to suggest which cell type effects reported in this manuscript are direct effects from loss of RANK within individual cell types versus indirect effects? RANK is expressed within immune cells and the intestinal epithelium, so its loss potentially affects the function of immune cells, function within the intestinal epithelium, and potentially the microbiota (which can influence the function and development of the intestinal epithelium). Are changes in immune invasion a result of changes in the intestinal epithelium, or changes in the immune cells themselves? Does RANK directly change development in the intestinal epithelium? Or do changes in the immune system or microbiota further influence it?
a. One idea is that potentially the authors' NF-KB transgenic could help resolve which cell types are directly experiencing a change in signaling in RANK mutants.
b. In the absence of additional data, it seems that the caveats at the end of discussion should be further expanded and

clarified. Or potentially other results from studying RANK or NF-KB within the immune system can be cited and discussed to exclude these possibilities?
6. Fig 5E-F is not clearly described and is overinterpreted. It is being used seemingly to claim that BEST4 cells are part of the secretory lineage while coyly phrasing that as a “closer relationship”, despite being insufficient evidence to claim a developmental relationship. It is not really required for the progression of the story, so I suggest removing it. If kept, it needs to be more clearly explained and not overinterpreted.
a. The results of an analysis to look at gene expression similarity are dependent on the set of genes whose expression is used to calculate similarity. However, this information (and the rationale for choosing the genes to compare) is not described in the text (and this analysis is completely left out of the methods section).
b. “Notably, within the secretory-associated branch, BEST4 cells displayed the strongest transcriptional similarity to goblet and tuft cells, suggesting a closer relationship with these specialist cells”. Though provocative, this analysis only shows gene expression similarity, which isn’t sufficient to claim a relationship between cell types — there are several famous cell types with shockingly similar transcriptional profiles, despite different developmental relationships (e.g. neural crest and mesodermal derivatives for many cell types such as cardiomyocytes, anterior and posterior notochord, or even intestinal enteroendocrine cells and pancreatic endocrine cells).
7. Figure 5C: This GO analysis is cited as evidence that larval and adult BEST4 cells have similar biology, but the top terms “multicellular organismal”, “developmental process”, and “homeostatic process” don’t seem very useful or to lend much credence to that idea. Though the results say that both populations express immune genes when calling this figure out, it does not seem that the immune system is a top GO term in the larval cells, based on the figure? This analysis should either be strengthened, have its description softened, or potentially be removed. To this reader, it did not seem to add much and could potentially be removed if the authors are trying to minimize revision work.
8. Important information is missing from the methods section.
a. There is no information about the feeding regime of the fish, which may potentially be important, since this is a study concerning gut biology in adults.
b. There is no description of how the anterior, mid, and posterior regions of the gut are delineated for this study, which may be important for future interpretation of the cell counts.
c. Most computational analyses are not described, including the trajectory analysis, GO enrichment, and transcriptional similarity analysis.

MINOR REQUESTED CLARIFICATIONS:

1. Figure 1D: Average expression cannot be below 0. This must be scaled expression?
2. Figure 1K-N: It appears that the morphology of the tuft / tuft-like cells change between these two timepoints, but this is not commented on in the results. Is this solely a reflection of the changing morphology of the intestine between these stages, or a more specific change within tuft / tuft-like themselves? Is it likely to reflect additional maturation of them that hasn’t happened by 6 days? It might be nice to address this directly in the results, if there is space.
3. Figure 2H: This analysis is in adult guts, correct? Not immediately clear from figure + figure legend, so suggest clarifying.
4. Figure 3E-F: Figure legend should clarify what staining is done here (LCP-1 IHC in orange, Alcian Blue in blue?)
5. Figure 4A-B: Which morphological features are used to define anterior/mid/posterior defined for this study?
6. The manuscript claims “We observed minimal induction of ILC2 activation markers in RANK mutants, supporting a requirement for tuft cells in ILC2 activation”. If this were true, since RANK mutants have fewer tuft cells, shouldn’t there be a downregulation of ILC2 markers in RANK mutants rather than a modest upregulation, since ILC2s could no longer be activated?
7. Figure 6I: Several individual data points for posterior rank mutants are below 0 and off of the plot, which seems that it must be inaccurate.

OPTIONAL SUGGESTIONS FOR THE AUTHORS THAT I THINK WOULD IMPROVE THE MANUSCRIPT:
1. Figure 3A-B’s presentation is somewhat difficult to interpret. There are a number of software packages designed for exactly what the authors want to do here: deconvolve changes in bulk RNAseq data based on a single-cell RNAseq reference. These software were recently reviewed in Xu et al Briefings in Bioinformatics 2025, with AutoGeneS being particular popular at the moment. If the authors are up to it, I suspect it would not be difficult to apply a published software package and might make for a much clearer result. Additionally, the authors immediately focused on changes in ILC3s, but the presented data seem to suggest that other leukocytes are also modified (e.g. macrophages?) and a proper analysis here would allow them to discuss those findings, potentially.

Version 1:

Reviewer comments:

Reviewer #2

(Remarks to the Author)
The authors have addressed our previous concerns and have added a statement outlining the study’s limitations. However, the current data and discussion remain insufficient to fully support the manuscript’s concept and title, and the revised version raises the following concerns.

Major points
#1:

The authors reported that rank-deficiency had modest effects on the abundance of pdia2+ tuft cells in the adult gut (Figure 5) and that RANK is necessary for the development of aloxa5-positive tuft cells in the adult gut, as stated in the subtitle. However, the authors only present data showing a pronounced decline in pou2f3-positive cells in the rank-deficient larval gut. There are no corresponding data on pou2f3-positive cells in the rank-deficient adult gut.
This inconsistency is confusing and may mislead readers into concluding that RANK affects both tuft cell lineages, based on the data shown in Figure 3, as noted in point 4 of our previous major comments. To clarify this point, the authors should include data on the number of pou2f3-positive cells in the rank-deficient adult gut (e.g., in Figure 3).
In addition, F-actin is not a specific marker for tuft cells, and the data presented in Figure 3I are difficult to interpret.

#2
Figure 4 shows leukocyte infiltration in the gut, accompanied by an inflammatory signature. This raises the possibility that inflammation contributes to the altered development of tuft cells and BEST4 cells. The authors should rule out the possibility that the inflammatory environment, characterized by leukocyte infiltration and upregulation of inflammatory signature genes following rank deletion in zebrafish, indirectly affects the development of these cells. Otherwise, there remains limited evidence that RANK signaling controls the lineage of intestinal epithelial cells.
It is well established that inflammatory conditions can influence lineage-specific differentiation. For instance, cytokines such as IL-13 are known to positively regulate tuft cell expansion, whereas IFN-γ or TNF could potentially alter type 2 immunity by inhibiting the expansion of IL-4/IL-13-producing cells.
To address this, the authors are encouraged to assess which inflammatory signals are activated under steady-state intestinal conditions (Fig. 4A) and evaluate how blocking these signals affects the development of both tuft cells and BEST4 cells.

#3
In Fig. 7A-D, the authors showed that exposure to Vc increased the number of cftr-positive cells and interpreted this as an expansion of BEST4 cells. However, cftr does not seem to be a specific marker for BEST4 cells. To support the conclusion, the authors should perform staining of the intestine with ‘best4’ as shown in Fig. 6I and 8G in order to properly identify BEST4 cells.
Alternatively, if the BEST4 cell specification is not provided, the authors should revise their interpretation to state that Vc exposure induces cftr expression in the intestinal epithelial cells.
In case best4-expressing BEST4 cells are indeed expanded following Vc exposure, it would also be important to assess whether this expansion is impaired in rank KO zebrafish.

#4
It would be valuable for the authors to also present scRNA-seq data from both WT and rank-KO zebrafish, as they have already compared gene expression profiles of infiltrating leukocytes in these groups in Figure 4A-B. If available, these data should be used to demonstrate how rank deletion alters the composition of intestinal epithelial cells.
Such analysis would help clarify how RANK signaling regulates lineage specification and differentiation of both tuft cells and BEST4 cells (i.e., if rank-deletion in zebrafish would affect the maintenance of precursor cells), as we requested in point 5 of our previous major comments. In addition, this data would provide insight into leukocyte populations infiltrating the intestine, as noted in point 6 of our previous major comments.

#5
The authors state in the newly added section, ‘Limitations of our study’, that they were unable to identify which cell type-specific RANK signaling is essential for the development of tuft cells and BEST4 cells.’ However, as shown in Supplementary Figure 3, the authors have already detected RANK-dependent NF-kB-activated cells in the gut tissues. At a minimum, the authors are encouraged to provide a histological characterization of these RANK-dependent NF-κB–activated cells by detecting GFP signals, which may be involved in regulating the differentiation of tuft cells and BEST4 cells.
Alternatively, the authors are encouraged to use the existing scRNA-seq data to identify the cell type in which NF-κB, a major downstream signaling pathway of RANK, is highly active in a RANK-dependent manner, by comparing datasets from WT and rank-KO zebrafish.

Minor points
#1
In Fig. 8B, the authors show only cropped images of the rank-GFP signal. The authors are encouraged to display images at the same scale as Anxa4.

#2
In Fig. 3A, the authors should more precisely indicate the BEST4 cell population in the plot by adding an arrow.

Reviewer #3

(Remarks to the Author)
I co-reviewed this manuscript with one of the reviewers who provided the listed reports. This is part of the Nature Communications initiative to facilitate training in peer review and to provide appropriate recognition for Early Career Researchers who co-review manuscripts.

Reviewer #4

(Remarks to the Author)
The authors have made laudable efforts in their revisions to correct issues raised by the reviewers through the addition of new analyses and data, but also modifying language to describe their results. Several of the added analyses and discussion are extremely nice and, to my eye, improve the manuscript. Hopefully, the authors agree. This is a great paper, and has addressed my concerns. There are still a couple of remaining issues of language that I will mention below that I think the authors should consider as they move through the publication process, since they would still have a chance to update them.
1. Line 346-347: This result is interpreted as indicating that BEST4 cells are important for response to Vibrio, but in rank mutants, BEST4 cells and tuft cells are compromised. It seems that the result would be more accurately described: ", suggest that BEST4 or tuft cells are important for managing Vibrio intestinal colonization"
2. I think the main text description of Fig 6K is still not accurate with regards to the analysis performed. See suggested revisions below in point 6.
3. The new analysis comparing zebrafish and mammalian tuft cell subtypes is awesome. This is up to the authors, but do you think it might be clearer to choose names for them in zebrafish that are not the same as mammals, since your analysis suggests that they are not direct equivalents? See Point 2 below. (THIS IS OPTIONAL AND AT AUTHOR PREROGATIVE)
4. Couple of typos that I happened to notice:
a. Line 305: give -> given
b. Fig 3C, y-axis legend: Bodly > Body
c. Fig 1D, legend still says "Avg. Exp"

Response to author's point-by-point:
1. The added discussion of mammalian results suggesting that two tuft cells are actually different maturation states of a single cell population are well done.
2. The analysis to compare zebrafish tuft cell types is really nicely done, and I think probably extremely informative. Given that this study is establishing the existence of these two zebrafish cell types, I think it's really valuable to identify how they relate to tuft cell types in other animals. For the authors to consider:
a. Given that your results suggest that the 2 tuft-cell populations that you're studying in zebrafish may not be equivalents to the 2 tuft-cell populations or maturation states that exist in mammals, is it worth changing their naming from "tuft 1" and "tuft 2" in your manuscript to something else? "tuft A" and "tuft B" or "tuft pdia2+" and "tuft alox5+"? Since I expect you will be working on these populations for some time, it might help prevent a common type of confusion that you would have to work against – the automatic assumption that your ZF Tuft-1 is the same as the mouse tuft-1. (I do not think the two zebrafish tuft cell populations are any less interesting if they do not perfectly correspond to two mammalian types.)
b. Lines 69 – 75: The use of "like mammals" in these sentences (especially for "resolved into two distinct subtypes" suggests to me here that you're going to make the argument that these cell subtypes are equivalent across animals, which might not be the case? Depending on how you'd like to present this, your readers might find the manuscript clearer
3. I think these modifications more accurately describe your study's impressive phenotypes.
4. I am fine with this.
5. I think the changes made to results & discussion with regards to this point have helped clarify the limitations of the approach (which does not decrease the value of the results whatsoever).
6. I am fine with the authors' decision to keep this analysis in their manuscript, and it does provide valuable information. The addition of the results from other groups is also warranted and helpful. However, I think the language is still somewhat mixed up about developmental relationships and transcriptional similarity, which aren't equivalent. The analysis here provides evidence that BEST4 cells might play a role more akin to other secretory cell types, since their transcriptional profile is more similar to other secretory cell types, but doesn't provide evidence that BEST4 cells have secretory origins (though the added references suggest that they do). I think the use of the term "relationship" in this paragraph is vague and confuses the two points, and I think it would be better revised to reflect the analysis done. The authors should feel free to re-write as they wish, but here is a suggested revision that I think more accurately describes your analysis:
a. 307: "their relationship to other intestinal cell types remains unclear, with more recent work" -> ", more recent work"
b. 311-312: "providing a quantitative measure of their relatedness" -> "providing a quantitative measure of their transcriptional similarity"
c. 315: "Our positioning of BEST4 cells within the secretory branch matches the observation" -> "Our positioning of BEST4 cells within the secretory branch shows these cells have a more similar transcriptional profile, and suggests that BEST4 cells may occupy a similar supportive or immune-related functional role in the intestine. This aligns with recent observations that"
7. This analysis is very cool and much more supportive of your points than the original one.
8. The more complete methods section is appreciated (and important!).

Version 2:

Reviewer comments:

Reviewer #2

(Remarks to the Author)
The authors have mostly addressed our concerns, which is appreciated. However, two points (raised in the previous round) remain.
Response to the authors' point-by-point:
#1 (point-by-point #2 in the previous version)

I appreciate that the authors have included a discussion of the potential contributions of immune-derived signals and

leukocyte–epithelium interactions as a limitation, which is helpful. It would further improve clarity to note that the observed epithelial phenotypes could, at least in part, be influenced by the inflammatory environment, and that a direct causal role of RANK signaling in epithelial lineage specification therefore remains to be fully established.
#2 (point-by-point #3 in the previous version)

The authors provide compelling evidence that cftr expression is highly enriched in BEST4 cells under homeostatic conditions, and the cited organoid studies further support a key functional role for CFTR in BEST4 cell–mediated responses to enteric pathogens. These data suggest that BEST4 cells represent a major population mediating CFTR-dependent fluid efflux.

However, it remains unclear whether cftr retains full specificity as a marker of BEST4 cells under infection conditions. While CFTR-dependent responses appear to be functionally restricted to BEST4 cells, this does not exclude the possibility that cftr expression may be modulated in other epithelial populations upon infection. Therefore, an increase in cftr-positive cells following Vc exposure may reflect either expansion of BEST4 cells or changes in cftr expression.

Given that the conclusion of increased BEST4 cells upon Vc infection is relatively novel, careful validation and interpretation are important. In this regard, confirming BEST4 cell identity using best4 expression would further strengthen the evidence for BEST4 cell expansion, as shown by the best4 probe in Figures 6I and 8G and in the cited organoid studies (PMID: 40010349).
The authors now state that Vc exposure increases the number of cftr-expressing epithelial cells, which is helpful. However, this statement is followed by the interpretation that this reflects an expansion of BEST4 cells. Given that cftr may not be a fully specific marker under infection conditions, this interpretation could be somewhat overstated.

It would improve clarity to more clearly separate the observation (increase in cftr-positive cells) from the inference (BEST4 cell expansion), or to slightly temper the wording in both the abstract and the main text to avoid overinterpretation. Addressing or clarifying this point would improve the precision of the conclusions.

Reviewer #3

(Remarks to the Author)
I co-reviewed this manuscript with one of the reviewers who provided the listed reports. This is part of the Nature Communications initiative to facilitate training in peer review and to provide appropriate recognition for Early Career Researchers who co-review manuscripts.

Reviewer #4

(Remarks to the Author)
The authors have satisfied my (few) remaining comments. I also agree with their responses to the other reviewers. While several of the proposed experiments are excellent lines of inquiry, to me they seem to be the beginning of entirely new studies, rather than being necessary for this already valuable study. The authors explicitly point out the limitations of their genetic tools and the need for additional future work to further address these questions; I think we should accept these already very informative data as-is, and hopefully additional studies will come out in the future that address some of the remaining questions.

Open Access This Peer Review File is licensed under a Creative Commons Attribution 4.0 International License, which permits use, sharing, adaptation, distribution and reproduction in any medium or format, as long as you give appropriate credit to the original author(s) and the source, provide a link to the Creative Commons license, and indicate if changes were made.
In cases where reviewers are anonymous, credit should be given to 'Anonymous Referee' and the source.
The images or other third party material in this Peer Review File are included in the article's Creative Commons license, unless indicated otherwise in a credit line to the material. If material is not included in the article's Creative Commons license and your intended use is not permitted by statutory regulation or exceeds the permitted use, you will need to obtain permission directly from the copyright holder.
To view a copy of this license, visit https://creativecommons.org/licenses/by/4.0/

REVIEWER COMMENTS
Reviewer #2 (Remarks to the Author):

In the manuscript "Rank-dependent control of tuft and Best4 cell development in the intestine," Willms and colleagues investigated how Rank regulates intestinal epithelial cell development in zebrafish. The authors demonstrated that systemic deletion of rank in zebrafish disrupts the maintenance of type 2-tuft cells and Best4 cells in the small intestine, altering the proportions of goblet cells. These findings illustrate how systemic Rank signaling impacts the development of intestinal epithelial cells and suggest potential physiological consequences of systemic rank deficiency.

However, a major limitation of this manuscript is the lack of investigation into the physiological relevance of the observed changes in epithelial cell development in rank-knockout zebrafish. Second, the data do not elucidate which downstream signaling pathways are involved in the maintenance of type-2 tuft cells and Best4+ cells. Moreover, some observations demonstrate a distinct role of Rank in the zebrafish, which is controversial in certain aspects, particularly the role of Rank in the development of Best4+ cells, which has not been observed in humans (DOI: 10.1016/j.stem.2025.02.003). However, as the authors in this manuscript only use full-body rank-knockout zebrafish, it remains unclear if the effects of Rank on Best4+ cell development are dependent on a direct role of Rank in the intestinal epithelial cells or secondarily from the effects of Rank in the other type of cells or other tissues.
We appreciate the reviewer's thoughtful assessment of our work and their supportive feedback. In our revised manuscript, we present new experimental data and text revisions that we feel address the major concerns raised in the initial review.
To examine the physiological consequences of RANK deficiency, we compared the ability of *Vibrio cholerae* to colonize the guts of *rank* mutant fish and their control wildtype siblings. We selected *Vibrio*, as the cholera toxin selectively targets CFTR+ cells in the gut, and zebrafish are and established model of *Vibrio* pathogenesis. Our new data (presented in revised Figure 7) show that *rank* mutants have a significantly impaired ability to clear pathogenic *Vibrio* relative to wildtype controls. As we also demonstrate that *Vibrio* promotes the intestinal accumulation of BEST4 cells (Figure 7A-D), and BEST4 cells are sensitive to diarrheagenic bacteria in human organoids (PMID: 40010349), we believe our new data provides valuable *in vivo* evidence that BEST4 cells contribute to host defenses against enteric pathogens.
Reviewer two also raises an important point about functional distinctions between systemic and cell-type specific RANK deficiency. We concur with the reviewer that these distinctions have implications for how we interpret our data. To properly acknowledge this point, we have reworked our Discussion in two key places. In the opening paragraph (lines 416 – 419), we have included the caveat that our work does not examine cell autonomous roles for RANK, and that these questions should be addressed in future studies. Separately, we have expanded the limitations section of the results to highlight instances where RANK signals from hematopoietic cells impact epithelial cell dynamics (lines 489 – 496). As reviewer two points out, communication between immune cells and epithelial cells may be important for some of the phenotypes described in our study. We appreciate this feedback from the reviewer, as it provides important context for readers.

RANK is well known to modulate immune cell functions, including self-tolerance in the thymus, lymph node organogenesis, and T cell activation, through signaling in thymic epithelial cells, lymphoid tissue inducer (LTi) cells, endothelial cells, and dendritic cells (doi:10.1038/16852; doi: 10.1101/gad.13.18.2412; DOI: 10.1016/j.immuni.2008.06.015). These immune-related alterations could happen in rank-deficient zebrafish, thereby likely influencing intestinal epithelial homeostasis as a secondary effect, since many previous reports demonstrated the maintenance of intestinal stem cells by immune cells. Thus, the changes observed in epithelial cell fate may result from indirect, immune-mediated effects rather than the direct effects of Rank signaling in the intestinal epithelial cells. The use of a full-body rank knockout model alone is insufficient to determine the specific role of Rank in the intestinal epithelial cells. To directly assess the epithelial-intrinsic role of Rank signaling, the authors should use either a conditional knockout strategy using a floxed rank allele with an intestinal-specific Cre driver or investigate zebrafish-derived intestinal organoid systems. These approaches would enable a more precise dissection of the cell-autonomous effects of Rank on intestinal epithelial cell development.
We agree that organoids and Cre recombinases would be useful tools to examine the impacts of epithelium-specific RANK ablation. Unfortunately, we are unaware of any successful gut organoids system for fish, and private discussions with a colleague working on this question suggest that there are substantial technical challenges to address before organoids will become routine. Likewise, we are not aware of a reliable CRE-based system for gene inactivation in the fish gut epithelium. As a result, we must regretfully conclude that these experiments are currently beyond the reach of our manuscript.

The authors need to address the following questions listed below.
Major comments;
1. The authors showed that rank expression on tuft cell precursors and type-2 tuft cells among their lineage cells. The authors should display the expression of rank in the entire intestinal tissue from scRNA-seq data, including all subtypes of intestinal epithelial cells, immune cells, and mesenchymal cells localized in the intestine. An overview of the rank expression in cells localized in the intestine will help elucidate in which cell types the impairment of Rank signaling occurs, as well as which cells may affect the fate of the intestinal cell lineage. Moreover, if the Rank signaling in the intestine affects Best4 cells' development, rank is also likely expressed on their lineage.

This is an important point that we had overlooked in our initial submission. The original manuscript had included a supplemental figure that used our scRNA-seq data to show *rank* expression in the fish gut, but we had neglected to point this out in the text. In our revised manuscript, we have embedded the data in Figure 3A. These new data show enriched expression of *rank* in progenitors and tuft cells, as well as mild expression of *rank* in goblet and BEST4 cells. We have also expanded the related text to state that we previously showed that commensal bacteria induce *rank* expression in larval BEST4 cells (PMID: 35108531). Combined, these findings support roles for *rank* in tuft cell development and are consistent with involvement of RANK in BEST4 cell development. We would like to express our gratitude to the reviewer for this suggestion.

2. It would be better to show the expression of spib, the master regulator of tuft cells in mice and humans (DOI: 10.1126/sciimmunol.ads5818; DOI: 10.1016/j.stem.2025.02.003), and sh2d6, as a marker for tuft-2 cells observed in mice, in Fig. 1D and F (https://doi.org/10.1016/j.immuni.2022.03.001).
To address these points, we have included new data looking at the expression of *sh2d6* and members of the *spi* transcription factor family in zebrafish guts. As predicted by the reviewer, our revised data demonstrate enriched expression of the zebrafish *sh2d6* ortholog in tuft cells (Revised Figure 1B). Looking at the Spi family, we mined our adult scRNA-seq data for the expression patterns of the four fish *spi* genes: *spi1a*, *spi1b*, *spi2* and *spic*. In each case, we find that gene expression is restricted to leukocytes in the gut (new data in supplementary figure 5A), most commonly in dendritic cells (new data in supplementary figure 5B). To support these findings, we used fluorescence *in situ* hybridization to examine expression of *cftr*, *spi2* (a zebrafish spib homolog) and *spic* in the fish gut. Our work shows that both *spi2* and *spic* are expressed in vasculature-associated cells, while confirming that *cftr* is restricted to the epithelium (new data in supplementary figure 5C-D), demonstrating that zebrafish BEST4 cells do not express *spi* homologs. Our findings also agree with a recent preprint from the Farrell group (PMID: 41446049) that also did not observe *spib* expression in zebrafish BEST4 cells. Based on these complementary findings from our group and the Farrell group, we have expanded our results section to suggest that unlike most mammals, zebrafish BEST4 cells are not marked by *spib* expression.

3. In the last sentences on page 5 and Fig. 1J, the authors mentioned that 'We found that alox5a-positive cells preferentially accumulated at the villus base,' however, the images in Fig. 1H show the localization of alox5a-positive cells in the apical site of villi. Usually, type-2 tuft cells localize in the tip of the villi as more differentiated tuft cells, as shown in the reference paper (https://doi.org/10.1016/j.immuni.2022.03.001). The authors need to clarify these data.
This is an important point that we have addressed with additional data and with added text. As the reviewer points out, the image in Figure 1J does not show basal *alox5a*+ cells. When we wrote the first draft of the manuscript, we selected this image as a representative example (from a total of 47 separate images) of how both *alox5a*+ and *pdia2*+ cells could be found along the entire villus axis. In the revised manuscript, we provide additional images (new data in supplementary Figure 2) that demonstrate an example where *alox5a*+ cells preferentially accumulate at the villus base. We like this representation of the data as it effectively captures the somewhat stochastic distribution of tuft cells along the villus axis. Prompted by a suggestion from reviewer four, we have also extended the discussion (lines 420 – 435) to compare the distribution of fish tuft cells to their mammalian counterparts.

4. In Fig. 2G, the authors showed that the pou2f3+ cells (pan-tuft cell marker) are barely detectable in rank KO zebrafish, suggesting that Rank signaling is essential for the development of whole tuft cells, including both type-1 and type-2; however, in Fig. 2C-E, the number of pdia2+ cells (type1 tuft cell marker) are comparable between WT and rank KO fish, implying that Rank signaling is not essential for development of type-1 tuft cells. The authors should reconcile these conflicting data.
Here, reviewer two raises an interesting point that we had also discussed internally. In Figure 3F and G of the revised manuscript, we show that, as expected, loss of RANK resulted in diminished expression of the *rank*:*GPF* reporter in adult and larval intestines. In Figure 3G, we also show that loss of RANK resulted in a pronounced decline in the number of *pou2f3*-positive cells in the larval gut. As the reviewer points out, this is in apparent contrast to the modest effects of RANK deficiency on the abundance of *pdia2*+ tuft cells in the adult gut. Although the reasons for these differences are not yet clear, we believe that they likely reflect the distinct developmental stages under examination, namely larvae and adults. At six days post-fertilization, the larval gut has only been fully open for approximately 48h. As a result, many intestinal epithelial cells have only recently encountered microbial factors. Additionally, it remains unclear to what extent ILCs populate the larval gut and how active those ILCs are. There is also very little experimental evidence to suggest that larval guts are populated by lymphocytes or dendritic cells. In this situation with a relatively naïve immune system and a simplified gut epithelium, loss of RANK leads to a pronounced loss of *pou2f3*+ cells. In adults, the intestine is a larger, more elaborate structure that hosts a distinct microbiota, and interacts with a complete repertoire of active myeloid and lymphoid lineages that include ILCs, mature B and T cells, dendritic cells and type1/2 macrophages. In this situation, loss of RANK has more modest effects on the abundance of *pou2f3*+ cells. We believe that the considerable cellular differences between the larval and adult gut may impact the responses of the two structures to RANK deletion.

5. Fig. 2D-F shows that rank-deletion causes the disapperance of rank-GFP-positive cells, which presumably include both her6, her9-expressing precursor cells and differentiated tuft cells. It appears that Rank signaling is

essential not only for tuft cell development but also for maintaining those precursor cells. The authors need to argue the results more precisely.

Reviewer two makes an interesting suggestion that we had not addressed in our initial submission. As RANK deficiency leads to a near complete loss of *rank*:*GFP*-positive cells, it is interesting to speculate that the mutation also affects progenitor cells, particularly given the pronounced expression of *rank* within the adult progenitor compartment. However, we are hesitant to make that claim at this point, as we have not provided rigorous proof that the reporter is fully expressed in, and marks, tuft progenitor cells. For that reason, we feel it is more appropriate to not make claims about effects of RANK deficiency on progenitors at this point. Nonetheless, we agree with the reviewer that this observation merits more detailed examination in the future.

6. The authors characterized leukocytes localized in the gut in Fig. 3. As observed in the whole body Rankl and Rank knockout mice (doi:10.1038/16852; doi: 10.1101/gad.13.18.2412), RANK-RANKL signaling controls lymph node organogenesis, extramedullary hematopoiesis. Thus, the different characters and the accumulation of immune cells in the intestine of rank-KO zebrafish may derive from dysregulation of immune cell development in lymphatic tissues. The author mentioned that rank deficiency shifts the hematopoietic compartment to a pro-inflammatory state. It would be meaningful if the authors addressed whether the intestinal tissues suffer from tissue damage, tissue inflammation, or cause intestinal barrier breakdown. Do rank KO zebrafish have higher sensitivity to any experimental colitis?

In response to these questions, we examined tissue architecture by H&E staining of *rank* mutants and their wildtype siblings in the absence of any damaging challenges. Additionally, we tested the effects of DSS exposure on *rank* mutant larvae as a measure of the ability of those mutants to respond to colitogenic stress. In brief, we did not detect any obvious intestinal defects in the mutants, and we found that mutants survived DSS exposure as effectively as wildtype controls. These data suggest that the epithelial barrier remains intact in *rank* mutants. However, we did observe effects of RANK deficiency on host clearance of *Vibrio* (see response to point 9), suggesting that *rank* mutants are impaired in their ability to clear pathogenic bacteria.

Moreover, although the author's subtitle for this section is 'RANK deficiency causes leukocyte accumulation in the gut,' the number of Lcp1+ cells seems only subtly different between WT and KO zebrafish, even though there is a significant difference between samples. It would be better to change the title towards a direction that more emphasizes the proinflammatory state of infiltrated immune cells. The authors need to investigate which cell types, such as T cells, B cells, monocytes/macrophages, granulocytes, and dendritic cells, are differentially accumulated in the intestine.

Reviewer two is correct that the initial title did not effectively capture the central message of data in this figure. In our revised manuscript, we have changed the relevant section title to "RANK Deficiency Impacts the Functional State of Gut-Associated Leukocytes". We feel this title more accurately represents the results and are grateful to the reviewer for this suggestion. We concur that it would be interesting to examine effects of RANK deficiency on the abundance of individual immune cell types. However, we are very hesitant to start such a complex undertaking at this point. As both reviewers point out, and as clarified in our revised manuscript (lines 224 – 229), we only see a mild accumulation of Lcp-1+ cells in the fish gut. As we lack antibodies or cell type-specific reporter fish for most gut associated cell types (e.g. ILC2s, ILC3s, dendritic cells, B cells, Macrophages), we feel that the time required to complete this experiment would go far beyond the scope of the current manuscript.

7. Fig.4A shows that the cell localization of alox5a-positive and rank-positive cells in the WT. Some of the cells localize close to the muscle layer, suggesting that there is a possibility that those positive cells are not epithelial cells, but other cell types localized in the lamina propria, such as immune cells or mesenchymal cells. To show the exact localization of positive cells and determine the cell type of those positive cells, the authors should co-stain the tissue with F-actin or an epithelial cell marker such as Epcam.

This is an interesting suggestion that we had not considered previously. To address this point, we performed a series of experiments where we used the 2F11 antibody to stained larval intestines for Anx4a while also staining for *alox5a* or *pdia2*. We selected 2F11 as it is an established marker of all secretory intestinal epithelial cells (PMID: 15689380), (new data in supplementary Figure 7A). Our work shows that *pdia2*+ (new data in supplementary Figure 7B) and *alox5a*+ cells (new data in supplementary Figure 7C) are also 2F11+, confirming that both cell types are epithelial. We would like to thank the reviewer for suggesting this experiment, as we feel it enhances the quality of our work.

8. In Fig. 5B, the authors should include ca7, the major Best4 cell marker, and spib, an important transcription factor (DOI: 10.1016/j.stem.2025.02.003).

In agreement with a recent preprint from the Farrell group (PMID: 41446049), we find that zebrafish BEST4 cells are marked by expression of *ca2* instead of *ca7* (Figure 6B of our revised manuscript). However, as pointed out by the authors of PMID: 41446049, it appears that *ca2* and *ca7* have similar functions and localizations, indicating a functional redundancy between the two genes.

9. What happens to the rank KO zebra fish after Vibrio cholerae challenge? Do the rank KO mice show difficulty in eliminating Vibrio cholerae and persistence of infection because of a lack of Best4+ cells as ionocytes? The authors should address the physiological outcome of the reduction of Best4+ cells in rank KO zebrafish.
This was a very interesting suggestion that we had not considered for our initial submission. To address this point, we studied the ability of RANK-deficient fish and their sibling controls to clear pathogenic strains of *Vibrio cholerae* after a 24h infection. Interestingly, we discovered that *rank* mutants were significantly impaired in their ability to clear *Vibrio* from their intestines. By five days after infection, we found that mutant intestines had approximately twice as many *Vibrio* as their wildtype controls, suggesting that loss of RANK impacts the ability of fish to manage a *Vibrio* infection. We would like to express our gratitude to the reviewer for this suggestion, as we feel it adds to our study, and to the field in general.

10. In lines 1-7, the authors discussed the discrepancy of the role of Rank signaling in the gut on Microfold cells and BEST4 cell differentiation between the data from the authors and the other papers. The authors need to point out that their data come from full-body knockout zebrafish, and this model might not focus on the role of Rank signaling in the intestine.
We concur with the reviewer and appreciate this important suggestion. As discussed above, we have addressed this issue by expanding the limitations section of the manuscript.

Minor points;
1. The legend for Figure 6D is missing. And the numbering of the figure legends in Fig.6D-H is wrong.
We have corrected these errors.

2. The author should explain or argue why the goblet cell signature gene expression is not altered, even though the number of the goblet cells is different between WT and KO in Fig. 2L and 6D.
This is an intriguing point that we had not considered in our initial data analysis. In our revised manuscript, we speculate that loss of RANK leads to enhanced specification of goblet cells without a parallel increase in goblet cell markers, suggesting diminished marker expression per goblet cell (lines 374 – 377). We are grateful to the reviewer for raising this intriguing point, as it suggests that RANK may have subtle but important effects on goblet cell function that merit consideration in future work.

3. The author should mention which statistic was used for Figs. 4 and 5, respectively.
We apologize for the oversight and have included the relevant information.

4. In the last line on page 18, ‘appear to’ is duplicated.
We have corrected this typographical error.

Reviewer #3 (Remarks to the Author):
I co-reviewed this manuscript with one of the reviewers who provided the listed reports. This is part of the Nature Communications initiative to facilitate training in peer review and to provide appropriate recognition for Early Career Researchers who co-review manuscripts.
We are grateful to reviewer three for their participation in the review process. All authors agree that the reviewer feedback substantially improved the quality of our manuscript and enhanced the impact of our work.

Reviewer #4 (Remarks to the Author):
OVERALL IMPRESSION
This is a nice manuscript that I think will be of broad interest and of impact within the intestinal biology community, with several novel results. I think that there are some results or analyses presented that are overinterpreted in the manuscript and should be revised. Additionally, I think that an expanded discussion that makes more clear some of the limitations of the conclusions of the manuscript is likely warranted. The Methods section currently omits several experiments or analyses.
We would like to express our gratitude to reviewer four for their supportive assessment of our study and for their feedback. We have reworked all sections of our manuscript to address points raised in the initial review and believe that the manuscript has improved considerably as a result.

SUMMARY OF RESULTS
Reems, McCabe, Jones, Schade, and Foley demonstrate that:
- Zebrafish have multiple types of tuft / tuft-like cells (alox5a and pdia2) that are found in both adults and larvae and have different distributions along the villus axis. These types express different genes, with the alox5a population expressing inflammatory genes linked to type two inflammation.

- RANK mutants exhibit gross morphological defects, including a shorter body length, shorter intestine, and exhibit scoliosis as they age.
- Adult RANK mutants have a complete loss of tuft / tuft-like cells, while larval RANK mutants have a severe reduction, though still maintain a small number of RANK:GFP+, pou2f3+ cells.
- RANK mutants result in increased expression of immune genes in bulk RNAseq, and show modestly increased infiltration of leukocytes in the intestine.
- RANK mutants have reduced alox5a tuft cells through the intestine and reduced pdia2 tuft cells in the anterior intestine.
- Adult zebrafish have BEST4 cells in all regions of the intestine, though they are enriched in the mid intestine, and along the entire length of the villus axis.
- cftr-positive cells increase after infection with Vibrio cholerae.
- RANK mutant intestines have reduced BEST4 cells in multiple regions of the intestine.
- RANK mutant intestines have reduced secretory cells, increased goblet cells, altered number of EECs, and reduced BEST4 cells

ISSUES THAT SHOULD BE ADDRESSED:

1. The authors propose that a common progenitor directly gives rise to the two types of tuft cells in zebrafish, and provide support in the form of a transcriptional trajectory. However, recent reports from humans and mice (Buissant des Amorie et al Nature Communications 2025) suggest that the two tuft cell types "represent successive stages of maturation drive by crypt-villus signaling gradients". This would be consistent with the different localization observed in zebrafish for these two subtypes, where the tuft-2 like cells are nearer the villus tips. Additionally, while rare, the authors do see some cells that co-express markers of both tuft cell types, which could be consistent with such a transition.

a. Ideally, the authors would conduct some kind of lineage tracing to resolve the differences. One possible experiment is that the authors could potentially use a transgenic marker of pdia2 tuft cells to confirm that alox5a tuft cells are not actually a further 'matured' state of these cells. Perhaps, however, there are simpler approaches that may address this issue?

b. Given that a lineage tracing experiment may be difficult in the timeframe of revisions of this manuscript, if not performed, it seems important to discuss the limitations of transcriptional trajectories, to discuss the mammalian lineage results that conflict with the proposed developmental relationships here, and to discuss any additional data that further suggests the two tuft-cell subtypes cannot be different maturation states of the same cell type (if it exists), and to suggest the need to future follow-up work.

Reviewer four raises an important idea that we had not considered in our initial submission. As the reviewer correctly points out, recent data suggest that mammalian tuft cells transition from progenitor through tuft-1 to tuft-2 states as they migrate from the crypt along the villus axis. It is interesting to speculate that fish Tuft-1 and Tuft-2 cells may correspond to similar maturation states. However, we feel our data do not adequately address that question for several reasons. From a structural perspective, fish intestines lack crypts, making it difficult to identify niches that maintain progenitor compartments. From our single cell data, we only observed co-expression of *pdia2* and *alox5a* in five of 635 tuft cells (with a Pearson correlation coefficient of -0.28), suggesting a lack of correlation between expression of the respective markers in tuft cells. From a comparative perspective (new data provided in new Figure 2), it appears that fish Tuft-1 and Tuft-2 are more closely related to mammalian tuft-p cells, and out trajectory analysis (Figure 1) suggests that the two markers represent distinct maturation states. We feel these observations are more consistent with a hypothesis that fish Tuft-1 and Tuft-2 cells are functionally distinct, mature cell types, but concur with reviewer four that cell marking studies are necessary to test that hypothesis. For that reason, we have carefully reworked the discussion (lines 420 – 435) to compare what we know about fish and mammalian tuft cells, elaborate on key differences between the two systems, and emphasize the need for additional work to test the developmental relationship of the two cell types in fish. We are very grateful to reviewer four for raising this point as it adds important context and value to our data.

2. The manuscript suggests that the two tuft-cell subtypes in zebrafish mirror the division of function of tuft-cell subtypes in mammalian systems. This is only supported with a handful of key genes, though. This point would be MUCH stronger if a broader comparison of their transcriptomes were performed — which seems in the authors' wheelhouse, based on their cell-type comparisons in Fig 5E-F. This would also strengthen their section in the discussion describing the relative cellular profiles between zebrafish and mammals and enable a stronger description of which features of mammalian tuft cells are best modeled within the zebrafish, which seems like a key point this manuscript would want to make if it is establishing that there are similar subtypes in zebrafish.

We appreciate this suggestion from reviewer four and feel that it adds to the question raised in point one above. In new data presented Figure 2, we examine the mouse tuft cell transcriptome (Figure 2A), and show that, like fish, mouse tuft cell progenitors are marked by enhanced expression of the *Rank* gene (Figure 2B). We also establish that the top eleven markers of fish tuft cells are expressed in mouse tuft cells, including the mouse orthologs of *Rank*, *Alox5a*, *Anxa4*, *Myb* and *Foxp1* (Figure 2C). Moving beyond individual markers, we use integrated data analysis (Figure 2E) to establish broad cross-species mixing of gene expression patters (Figure 2F). Interestingly, our comparisons show that fish Tuft-1 and Tuft-2 cells are most closely related to the mouse tuft-p cell type, suggesting that fish tuft cells may be most closely related to an ancestral tuft cell that diversified during mammalian evolution (Figure 2D). We had not considered this approach to our data, but we feel that it provides useful insights into the relationship between mammalian and zebrafish tuft cells, and we would like to thank the reviewer for proposing this line of investigation.

3. The manuscript overinterprets the necessity of RANK in some places. Tuft and BEST4 cells are reduced in number in RANK mutants, but not eliminated by any means. Thus, it seems that descriptions of 'necessity' or 'essentiality' are not accurate since these cell types are still capable of developing in the absence of RANK (e.g. subtitles "RANK is Necessary for Development of alox5a-positive Tuft Cells" and "RANK is Necessary for BEST4 Cell Development", as well as text references "requirement for RANK in the development of immune regulatory tuft cells", "confirming an essential role for RANK in the development of Anxa4-positive cells", and potentially others – the authors should do a comprehensive sweep). It would be more appropriate to suggest that RANK "plays a role" or "modifies development" or "influences development". Similarly "control" in the title might be too strong of a term for a partial effect?

We concur with the reviewer on this point. We have carefully re-examined the manuscript with this thought in mind and changed wording throughout that our work supports a role for RANK in the specification of intestinal epithelial cell types, without overstating any essential involvements.

4. Figure 2G-H: it's unclear why the assay presented switches from pou2f3+ cells to f-actin+ cells between the larva and adult. While it's interesting to also show the f-actin results (which I suppose suggest that tuft cells don't differentiate properly in RANK mutants?), since adults have also been stained for pou2f3 (Fig 1L, N), I think they should be quantified and displayed in place of 2H, though I would support keeping the f-actin result also in the main figure as a later panel. Currently, this gives the impression that tuft cells are completely lost in RANK mutants, which does not seem to be true, since there are several alox5a or pdia2 cells still in the animal (Figure 4).

For Figure 2, we found that f-actin effectively stained rank:GFP+ cells in adult guts, but less so in larvae. This is most likely due to the fact that the large, elaborate villi in adult intestines are more accessible for sectioning and staining that the smaller, less obvious structures in larval intestines. We feel that the f-actin stain merits inclusion, as it reveals a defining structural feature of tuft cells. As our single cell studies had identified two tuft cell subtypes (Figure 1), and our transcriptomic work had already shown that RANK deficiency led to a significant decline in *pou2f3* expression (Revised Figure 3), we elected to characterize the consequences of the *rank* mutation for the abundance of *alox5a* and *pdia2*-positive cells (Revised Figure 4) instead of the less specific characterization of *pou2f3*-positive cells. For those reasons, we respectfully argue that the current organization of Figure 3 (Figure 2 from the initial submission) is best left in its current form.

5. Do the authors have any data to suggest which cell type effects reported in this manuscript are direct effects from loss of RANK within individual cell types versus indirect effects? RANK is expressed within immune cells and the intestinal epithelium, so its loss potentially affects the function of immune cells, function within the intestinal epithelium, and potentially the microbiota (which can influence the function and development of the intestinal epithelium). Are changes in immune invasion a result of changes in the intestinal epithelium, or changes in the immune cells themselves? Does RANK directly change development in the intestinal epithelium? Or do changes in the immune system or microbiota further influence it?

a. One idea is that potentially the authors' NF-KB transgenic could help resolve which cell types are directly experiencing a change in signaling in RANK mutants.

b. In the absence of additional data, it seems that the caveats at the end of discussion should be further expanded and clarified. Or potentially other results from studying RANK or NF-KB within the immune system can be cited and discussed to exclude these possibilities?

Here, reviewer four raises a point that aligns with comments from reviewer two. The work in this manuscript focussed on the consequences of systemic RANK deficiency for gut homeostasis, with noted impacts on leukocytes, tuft cells and BEST4 cells. In the absence of cell type-specific mutations, we cannot yet assess the extent to which the reported phenotypes are cell-autonomous, or reflect impaired cell-cell interactions in the gut. As a result, we reworked the Introduction, Results, Discussion and Limitations sections to clarify the value of lineage-restricted RANK inactivation from future work. We appreciate this suggestion from both reviewers. We feel these improvements to the text provide useful contextual information to readers and identify potentially productive areas of investigation for the future.

6. Fig 5E-F is not clearly described and is overinterpreted. It is being used seemingly to claim that BEST4 cells are part of the secretory lineage while coyly phrasing that as a "closer relationship", despite being insufficient evidence to claim a developmental relationship. It is not really required for the progression of the story, so I suggest removing it. If kept, it needs to be more clearly explained and not overinterpreted.

a. The results of an analysis to look at gene expression similarity are dependent on the set of genes whose expression is used to calculate similarity. However, this information (and the rationale for choosing the genes to compare) is not described in the text (and this analysis is completely left out of the methods section).

b. "Notably, within the secretory-associated branch, BEST4 cells displayed the strongest transcriptional similarity to goblet and tuft cells, suggesting a closer relationship with these specialist cells". Though provocative, this analysis only shows gene expression similarity, which isn't sufficient to claim a relationship between cell types — there are several famous cell types with shockingly similar transcriptional profiles, despite different developmental relationships (e.g. neural crest and mesodermal derivatives for many cell types such as

cardiomyocytes, anterior and posterior notochord, or even intestinal enteroendocrine cells and pancreatic endocrine cells).

We agree with reviewer four that the language related to these panels had room for improvement. However, we feel that the relevant data are worthy of inclusion in the manuscript. We are at the very early stages of functionally characterizing BEST4 cells, including understanding how BEST4 cells relate to all other epithelial cell types. Early work (PMID: 31348891) suggested that BEST4 cells were absorptive cells that were most closely related to enterocytes, a cell type primarily associated with the acquisition and processing of nutrients. We feel our analysis is important as it raises the possibility that BEST4 cells are more closely related to goblet cells and tuft cells, epithelial specialists associated with immune regulation. We agree with the reviewer that these findings are not in and of themselves adequate to demonstrate relatedness and we concur that they may be another instance of developmentally unrelated cells with similar transcriptional programs. However, we believe our analysis is supported by recent data showing that rat BEST4 cells share a progenitor with tuft cells (PMID: 40371707) alongside in-depth transcriptomic work in a very recent preprint from the Farrell group that places BEST4 cell development within the secretory lineage (PMID: 41446049). To address this point, we have reworked the relevant text (Lines 307 – 318) to clarify that the pairwise comparisons were based on single cell-derived transcriptomes for each cell type and to place our work within the context of PMIDs 31348891, 40371707 and 41446049, while deferring to PMID: 41446049 as a more definitive computational analysis of BEST4 cell development. We feel this is a substantially improved representation of the data that should make our work more relevant to the field and appreciate the suggestions from reviewer four.

7. Figure 5C: This GO analysis is cited as evidence that larval and adult BEST4 cells have similar biology, but the top terms “multicellular organismal”, “developmental process”, and “homeostatic process” don’t seem very useful or to lend much credence to that idea. Though the results say that both populations express immune genes when calling this figure out, it does not seem that the immune system is a top GO term in the larval cells, based on the figure? This analysis should either be strengthened, have its description softened, or potentially be removed. To this reader, it did not seem to add much and could potentially be removed if the authors are trying to minimize revision work.

We concur with reviewer four that a rudimentary GO analysis is inadequate to compare larval and adult BEST4 cells, particularly given the inherent value of comparing the same cell in two very different developmental stages (the emerging intestine of a larva versus the more complex intestine of an adult). Inspired by reviewer four’s comments, we therefore elected to compare larval and adult BEST4 cells in considerably greater detail (lines 269-288). We first selected the top 50 markers of larval and adult BEST4 cells and asked how effectively they marked BEST4 cells across development. In this analysis (new data in Figure 7C), we showed that 24% of all markers retain their utility as BEST4 cell markers in larvae and adults, but also that many larval markers are minimally expressed in adult BEST4 cells, suggesting shifts in marker gene expression with development. Moving beyond markers, we used the top 200 BEST4 cell-associated genes from each developmental stage to define larval and adult BEST4 gene expression programs. Comparing the expression of each program in larvae and adults, we showed that the larval program effectively defines BEST4 cells in both larvae and adults (new data in Figure 7D, F and H). However, we also discovered that while the adult program clearly defines adult BEST4 cells (new data in Figure 7G), it also marked additional cell types in the larva (Figure 7E). Combined, the new data presented in Figure 7C-H indicate that the larval BEST4 gene expression program persists in adult BEST4 cells, but also that adult BEST4 cells acquire additional functions as the animal ages. We believe this is a valuable perspective on understanding BEST4 cell function *in vivo* and would like to express our gratitude to reviewer four for raising this point. Parenthetically, we note that the additional data led us to present the host-Vibrio interactions as a standalone figure. We feel this is a more effective encapsulation of our results that improves the flow of the manuscript.

8. Important information is missing from the methods section.
a. There is no information about the feeding regime of the fish, which may potentially be important, since this is a study concerning gut biology in adults.
b. There is no description of how the anterior, mid, and posterior regions of the gut are delineated for this study, which may be important for future interpretation of the cell counts.
c. Most computational analyses are not described, including the trajectory analysis, GO enrichment, and transcriptional similarity analysis.

We have updated all missing pieces of information, including feeding regime (lines 497 - 503), and a citation for Wang et al. (BMC Genomics 2010, PMID: 20565988) that details the morphological criteria used to define anterior, middle and posterior sections of the adult intestine. We have also provided links to scripts used for trajectory analysis and provided a detailed methods section for similarity analysis done between larval and adult BEST4 cells, and between fish and mouse tuft cells.

MINOR REQUESTED CLARIFICATIONS:
1. Figure 1D: Average expression cannot be below 0. This must be scaled expression?
Reviewer four is correct on this point, and we have corrected the image accordingly.

2. Figure 1K-N: It appears that the morphology of the tuft / tuft-like cells change between these two timepoints, but this is not commented on in the results. Is this solely a reflection of the changing morphology of the intestine

between these stages, or a more specific change within tuft / tuft-like themselves? Is it likely to reflect additional maturation of them that hasn't happened by 6 days? It might be nice to address this directly in the results, if there is space.
This is an interesting question, that partly relates to our comparisons between larval and adult BEST4 cells: Do tuft cells also mature with age? We speculate that this is quite likely the case. Larval and adult intestines are quite different in terms of microbiome composition, dietary needs, cell composition and structure, so it is likely that each cell type adapts, including morphologically, to their changing environments. However, we are hesitant to include such speculative statements in the manuscript at this point, as we feel that a more rigorous comparison of the intestines of larval and adult fish is worthy of follow-up examination in the future.

3. Figure 2H: This analysis is in adult guts, correct? Not immediately clear from figure + figure legend, so suggest clarifying.
We have improved the text in Figure 2H to clarify which work was done in adults and which work was done in larvae.

4. Figure 3E-F: Figure legend should clarify what staining is done here (LCP-1 IHC in orange, Alcian Blue in blue?)
We would like to thank the reviewer for catching this omission. We have now updated the legends to clarify what is being stained in the respective panels.

5. Figure 4A-B: Which morphological features are used to define anterior/mid/posterior defined for this study?
We have updated the results and materials and methods section to cite Wang et al. (BMC Genomics 2010, PMID: 20565988) for details on the morphological criteria used to define anterior (rostral intestinal bulb), middle (mid-intestine), and posterior (caudal intestine) intestinal segments

6. The manuscript claims "We observed minimal induction of ILC2 activation markers in RANK mutants, supporting a requirement for tuft cells in ILC2 activation". If this were true, since RANK mutants have fewer tuft cells, shouldn't there be a downregulation of ILC2 markers in RANK mutants rather than a modest upregulation, since ILC2s could no longer be activated?
Reviewer four is correct, and our initial presentation of the data did not accurately demonstrate this point. In our revised manuscript, we show that the classical ILC2 marker *il13* is one of the few downregulated genes in *rank* mutant zebrafish (Revised Figure 3B and accompanying text), confirming downregulation of key ILC2 markers in *rank* mutants.

7. Figure 6I: Several individual data points for posterior rank mutants are below 0 and off of the plot, which seems that it must be inaccurate.
This was an error that all authors missed in the initial draft. We have fixed the plot to correct this mistake. We are grateful to the reviewer for bringing this to our attention. We would also like to clarify that the error in the figure had no impact on the underlying statistics.

OPTIONAL SUGGESTIONS FOR THE AUTHORS THAT I THINK WOULD IMPROVE THE MANUSCRIPT:
1. Figure 3A-B's presentation is somewhat difficult to interpret. There are a number of software packages designed for exactly what the authors want to do here: deconvolve changes in bulk RNAseq data based on a single-cell RNAseq reference. These software were recently reviewed in Xu et al Briefings in Bioinformatics 2025, with AutoGeneS being particular popular at the moment. If the authors are up to it, I suspect it would not be difficult to apply a published software package and might make for a much clearer result. Additionally, the authors immediately focused on changes in ILC3s, but the presented data seem to suggest that other leukocytes are also modified (e.g. macrophages?) and a proper analysis here would allow them to discuss those findings, potentially.
I (EF) tried to download, install and run AutoGeneS on my computer. Unfortunately, I could never quite get it to install and run smoothly despite persisting for over a week – this may reflect a lack of technical proficiency on my part. Nonetheless, we agree with Reviewer four that the presentation of data in Figure 4A-B (Figure 3A-B of original manuscript) had room for improvement. For that reason, we presented the data in Figure 4B as a classical expression heatmap that is widely used in the study of single cell gene expression patterns. To orient readers, we also marked the expression patterns of multiple regulators of inflammation, so that readers can better understand which genes are being induced by RANK deficiency and where those genes are typically expressed. For example, readers can now quickly see that the ILC3 markers *nitr5* and *nitr4a* are significantly upregulated in *rank* mutants, while the tuft cell-responsive ILC2 marker *il13* is downregulated. We feel this makes the results easier to interpret than the original layout and would like to thank the reviewer for this helpful suggestion.

REVIEWER COMMENTS

REVIEWER #2 (REMARKS TO THE AUTHOR):
The authors have addressed our previous concerns and have added a statement outlining the study's limitations. However, the current data and discussion remain insufficient to fully support the manuscript's concept and title, and the revised version raises the following concerns.
We are grateful that reviewer two acknowledges that we have addressed concerns raised in the previous round of reviews, and we appreciate their ongoing efforts to ensure that our manuscript is of a sufficiently high quality.

Major points
#1:
The authors reported that rank-deficiency had modest effects on the abundance of pdia2+ tuft cells in the adult gut (Figure 5) and that RANK is necessary for the development of aloxa5-positive tuft cells in the adult gut, as stated in the subtitle. However, the authors only present data showing a pronounced decline in pou2f3-positive cells in the rank-deficient larval gut. There are no corresponding data on pou2f3-positive cells in the rank-deficient adult gut.
This inconsistency is confusing and may mislead readers into concluding that RANK affects both tuft cell lineages, based on the data shown in Figure 3, as noted in point 4 of our previous major comments. To clarify this point, the authors should include data on the number of pou2f3-positive cells in the rank-deficient adult gut (e.g., in Figure 3).
In addition, F-actin is not a specific marker for tuft cells, and the data presented in Figure 3I are difficult to interpret.
Reviewer two is correct that the inclusion of *pou2f3*+ cell counts in adult *rank* mutant guts will enhance the depth of our findings further. In our revised manuscript, we provide a new supplementary figure to address this question (new data in Supplementary Figure 5). As expected, we see a significant, but not complete loss of *pou2f3*+ cells in the intestines of adult zebrafish. These data align with our observations that loss of RANK leads to a significant decline in the number of *alox5a*+ cells in the adult intestine. We concur with reviewer two that this finding strengthens our work and appreciate the suggestion to include these data.
We also agree with the reviewer that F-actin alone is not a specific marker of tuft cells. However, as described in citations 25 and 26 (PMID: 9214549 and PMID: 3688463) tuft cells have a rather remarkable cytoskeletal architecture that includes actin-rich bundles at the cell periphery. We feel this is germane to our work, as we demonstrated previously that the larval fish intestine contains a rare population of epithelial cells with classic feature of tuft cell morphology, including dense actin-rich bundles at the cell periphery (Supplemental Figure 3D; PMID: 35108531). For those reasons, we feel that demonstration of a loss of F-actin rich, *rank* and *pou2f3*+ cells merits inclusion in this work.

#2
Figure 4 shows leukocyte infiltration in the gut, accompanied by an inflammatory signature. This raises the possibility that inflammation contributes to the altered development of tuft cells and BEST4 cells. The authors should rule out the possibility that the inflammatory environment, characterized by leukocyte infiltration and upregulation of inflammatory signature genes following rank deletion in zebrafish, indirectly affects the development of these cells. Otherwise, there remains limited evidence that RANK signaling controls the lineage of intestinal epithelial cells.
It is well established that inflammatory conditions can influence lineage-specific differentiation. For instance, cytokines such as IL-13 are known to positively regulate tuft cell expansion, whereas IFN-γ or TNF could potentially alter type 2 immunity by inhibiting the expansion of IL-4/IL-13-producing cells.
To address this, the authors are encouraged to assess which inflammatory signals are activated under steady-state intestinal conditions (Fig. 4A) and evaluate how blocking these signals affects the development of both tuft cells and BEST4 cells.
We agree with reviewer two that it will be of interest to test if inflammatory cues from gut-associated leukocytes impact cell dynamics in the intestinal epithelium of wildtype or RANK-deficient fish. For those

reasons and inspired by earlier suggestions from reviewers two and four, we had reorganized text in the introduction, results, and discussion to emphasize the importance of considering leukocyte-epithelium interactions in the evaluation of our data, and in the design of future studies. Separately, we had re-worked Figures 4A and B to demonstrate the expression patterns of leukocyte-associated inflammatory regulators that are affected by RANK deficiency. To strengthen this aspect of our work further, we have now provided a new data set (new data in supplementary table 1) that identifies all leukocyte-associated genes that are dysregulated in *rank* mutants. We feel the provision of these data better illustrates immune response genes that are affected by RANK deficiency, and we believe these data will be of interest to readers. We are grateful to reviewer two for their comments on this point, as it provides additional context to the body of work presented in this manuscript.

However, we must respectfully disagree with reviewer two that we should pursue this aspect of our findings further by blocking candidate mediators of leukocyte-epithelium interactors. We feel that properly characterizing interactions between any one hematopoietic cell type and intestinal epithelial cell dynamics requires a study as rich and detailed as the manuscript currently under consideration. As the reviewer can doubtlessly appreciate, an undertaking of this magnitude will require investments of time and labour that go far beyond the scope of this manuscript. From our perspective, addressing this question is best done in a separate follow-up study. We believe that our current approach of highlighting possible contributions of leukocytes to the novel phenotypes already described in this study, while acknowledging the need for cell-specific studies in the future, is the most appropriate avenue at this point, as it ensures that all readers are aware of the broader context of our findings.

#3

In Fig. 7A-D, the authors showed that exposure to Vc increased the number of cftr-positive cells and interpreted this as an expansion of BEST4 cells. However, cftr does not seem to be a specific marker for BEST4 cells. To support the conclusion, the authors should perform staining of the intestine with 'best4' as shown in Fig. 6I and 8G in order to properly identify BEST4 cells.

Alternatively, if the BEST4 cell specification is not provided, the authors should revise their interpretation to state that Vc exposure induces cftr expression in the intestinal epithelial cells.

In case best4-expressing BEST4 cells are indeed expanded following Vc exposure, it would also be important to assess whether this expansion is impaired in rank KO zebrafish.

Here, we must respectfully disagree with reviewer two. In our hands, *cftr* is an excellent marker of BEST4 cells in the adult intestine (e.g. dotplot in Figure 6B and appended feature plot in Figure A showing that *cftr* expression is restricted to BEST4 cells). Furthermore, our *in situ* hybridization work demonstrates a near-complete overlap of *best4* and *cftr* expression in the fish intestinal epithelium (e.g. appended *in situ* hybridization images in Figure B). Indeed, BEST4 cells are regularly described as BEST4/CFTR-high expressing cells (BCHE) elsewhere in the literature, and the immediate involvement of CFTR in BEST4 cell responses to diarrheagenic bacteria has been established in human intestinal organoids (PMID: 40010349). In that study, the authors explicitly stated that "BEST4/CA7^{+} cells are the only *CFTR*-high expressers in human small intestine" and the authors presented experimental evidence that CFTR function in BEST4 cells is directly responsible for organoid swelling induced by the cholera toxin. Given the BEST4 cell-specific expression of *cftr*, and the immediate role of CFTR in *Vibrio cholerae*-mediated intestinal pathology, we feel that it is appropriate to directly quantify the impact of *Vibrio* on *cftr*+ cells in our experiments. Nonetheless, and out of an abundance of caution, we also ensured that the relevant text confirmed that we observed increased abundance of *cftr*+ cells in infected guts ("In these experiments, we discovered that exposure to *Vc* led to a significant increase in the number of *cftr*-positive intestinal epithelial cells at both time points (Figure 7D)".

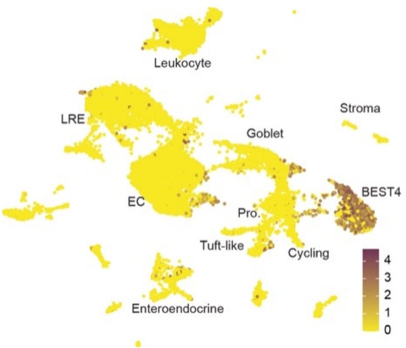

Figure A: Feature plot showing the expression of *cftr* in the adult zebrafish intestine, with all major cell types labelled.

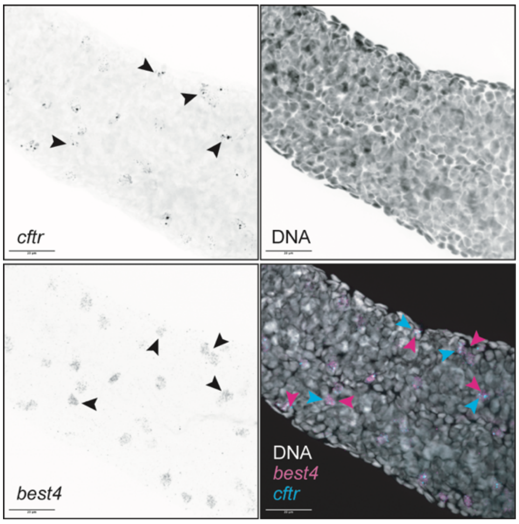

Figure B: Fluorescence *in situ* hybridization images showing overlapping expression of *cftr* and *best4* genes in a larval zebrafish intestine. In the false colored image, *best4* expression is in magenta and *cftr* is labelled in cyan. In each panel, arrowheads point to representative cells that are double-positive for *cftr* and *best4*.

#4

It would be valuable for the authors to also present scRNA-seq data from both WT and rank-KO zebrafish, as they have already compared gene expression profiles of infiltrating leukocytes in these groups in Figure 4A-B. If available, these data should be used to demonstrate how rank deletion alters the composition of intestinal epithelial cells.

Such analysis would help clarify how RANK signaling regulates lineage specification and differentiation of both tuft cells and BEST4 cells (i.e., if rank-deletion in zebrafish would affect the maintenance of precursor cells), as we requested in point 5 of our previous major comments. In addition, this data would provide insight into leukocyte populations infiltrating the intestine, as noted in point 6 of our previous major comments.

We concur with the reviewer that examining the consequences of RANK deficiency to the level of individual cells has merit, and we agree that these studies are a worthy follow-up from the data presented in our study. However, we feel it is also important to clarify that our study already addressed the reviewer's request to demonstrate how *rank* deletion alters the composition of intestinal epithelial cells. Specifically, we showed the effects of RANK deficiency on the intestinal epithelial abundance of tuft cells (Figures 3 and 5), BEST4 cells (Figure 8G-I), goblet cells (Figure 8D-F) and enteroendocrine cells (Figure 8G-I). We went beyond that by stratifying the impacts of RANK deficiency on enteroendocrine, BEST4, and tuft cell abundance by intestinal region, and we extend this study further by clarifying regional effects of RANK deficiency on the abundance of *aloxa5*+ and *pdia2*+ tuft cell subsets. We feel that such a direct and detailed quantification of epithelial cell type abundance provides a reliable measure of the consequences of *rank* deletion for intestinal epithelial cell type composition. We also examined the effects of RANK deficiency on the expression of leukocyte-associated

genes (Figure 4), and we showed that RANK loss diminished expression of ILC2 and DC markers, while enhancing expression of B cell and macrophage markers (Figure 4A-C and new data in supplementary table 1). We believe these studies provide actionable insights into candidate leukocyte populations infiltrating the intestine, and we look forward to testing this hypothesis in future work.
We agree with reviewer two that a detailed single cell transcriptomic comparison of RANK and wildtype intestines are a valuable tool to look even closer at how RANK deficiency affects all leukocyte lineages. However, we are of the opinion that such work, if performed properly, would amount to an entirely new study that would be poorly served by inclusion as a supplemental discovery in this manuscript. For those reasons, we respectfully suggest that the leukocyte-relevant data and accompanying text in the current manuscript are most effectively presented as is.

#5

The authors state in the newly added section, 'Limitations of our study', that they were unable to identify which cell type-specific RANK signaling is essential for the development of tuft cells and BEST4 cells.' However, as shown in Supplementary Figure 3, the authors have already detected RANK-dependent NF-kB-activated cells in the gut tissues. At a minimum, the authors are encouraged to provide a histological characterization of these RANK-dependent NF-κB–activated cells by detecting GFP signals, which may be involved in regulating the differentiation of tuft cells and BEST4 cells.
Alternatively, the authors are encouraged to use the existing scRNA-seq data to identify the cell type in which NF-κB, a major downstream signaling pathway of RANK, is highly active in a RANK-dependent manner, by comparing datasets from WT and rank-KO zebrafish.
This was an interesting suggestion from reviewer two that we had not previously considered. To address this question, we have undertaken additional in-depth analyses of genes that are downregulated within the intestinal epithelium of *rank* mutants. To this end, we used all significantly downregulated genes to define a RANK-deficiency gene expression module. We then plotted the expression pattern of this module on our adult single cell gene expression atlas (new data in Figure 3N, see below) and quantified the module score for each epithelial cell type on a per-cell basis (new data in Figure 3O, presented below), as well as the average module score for each epithelial lineage (new data in Figure 3P, see below). We are pleased to share that this approach clearly demonstrates that loss of RANK significantly affects expression of genes associated with two epithelial lineages. Specifically, tuft cells showed marked enrichment of the RANK-deficiency gene expression module, while BEST4 cells show a more moderate enrichment (new data in Figure 3N-P). We are grateful to the reviewer for suggesting this approach, and we are gratified that this new approach overlaps with all other data in our manuscript pointing to links between RANK signaling and the development of tuft and BEST4 cells.

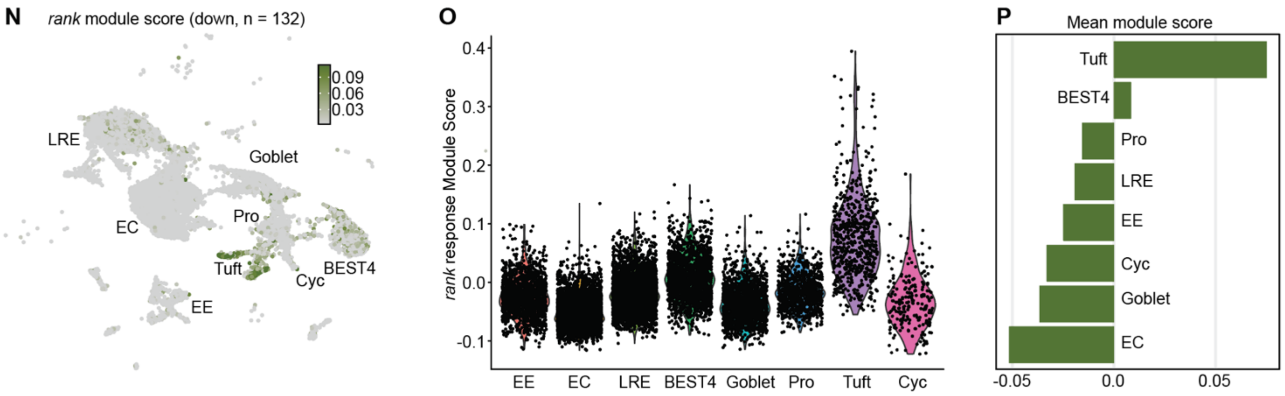

New data from Figure 3. Panel N shows the cell type distribution of the RANK-deficiency gene expression module in the adult zebrafish intestine, while O graphs the module score for each individual cell, and P shows the mean module score for each lineage. These new data confirm that loss of RANK primarily suppresses expression of tuft cell-associated genes but also diminishes expression of BEST4 cell-associate genes.

Minor points#1

In Fig. 8B, the authors show only cropped images of the rank-GFP signal. The authors are encouraged to display images at the same scale as Anxa4.

Our intent with the experiment represented in Figure 8A-C was to test for general effects of RANK deficiency on the epithelial abundance of Anxa4+ cells. However, we agree that a single magnified panel of a *rank:GFP+* cell does not fit particularly well in that context. For that reason, and to retain a focus on Anxa4+ cell counts, we have re-organized Figure 8 by removing the insert panel. We feel this is a cleaner, more streamlined description of the phenotype in question, and appreciate the comment from reviewer two.

#2

In Fig. 3A, the authors should more precisely indicate the BEST4 cell population in the plot by adding an arrow.

We are grateful to the reviewer for this suggestion and have made the suggested improvement to the figure.

REVIEWER #3 (REMARKS TO THE AUTHOR):

I co-reviewed this manuscript with one of the reviewers who provided the listed reports. This is part of the Nature Communications initiative to facilitate training in peer review and to provide appropriate recognition for Early Career Researchers who co-review manuscripts.

We appreciate the contributions of reviewer three to our work. We feel the comments from both rounds of reviews have elevated our findings and are grateful to the reviewer for their time and input.

REVIEWER #4 (REMARKS TO THE AUTHOR):

The authors have made laudable efforts in their revisions to correct issues raised by the reviewers through the addition of new analyses and data, but also modifying language to describe their results. Several of the added analyses and discussion are extremely nice and, to my eye, improve the manuscript. Hopefully, the authors agree. This is a great paper, and has addressed my concerns. There are still a couple of remaining issues of language that I will mention below that I think the authors should consider as they move through the publication process, since they would still have a chance to update them.

We would like to start by expressing our gratitude to reviewer four. Feedback from all the reviewers has clearly improved the quality of our work but also provided us important food for thought as we consider next steps in this project. We were particularly gratified to see the reviewer's enthusiasm for our study. We feel our work has value for the field, and we feel the remaining suggestions from reviewer four enhance the contextualization of our work.

1. Line 346-347: This result is interpreted as indicating that BEST4 cells are important for response to Vibrio, but in rank mutants, BEST4 cells and tuft cells are compromised. It seems that the result would be more accurately described: ", suggest that BEST4 or tuft cells are important for managing Vibrio intestinal colonization"

We agree with this suggestion and have reworked the text accordingly.

2. I think the main text description of Fig 6K is still not accurate with regards to the analysis performed. See suggested revisions below in point 6.

This was an important point that we address in detail below.

3. The new analysis comparing zebrafish and mammalian tuft cell subtypes is awesome. This is up to the authors, but do you think it might be clearer to choose names for them in zebrafish that are not the same as mammals, since your analysis suggests that they are not direct equivalents? See Point 2 below. (THIS IS OPTIONAL AND AT AUTHOR PREROGATIVE)

We are glad that reviewer four appreciated the comparative studies. We wholeheartedly agree that this new figure adds important details to the overall study. We also agree with reviewer four that these findings suggest that it is best to use distinct names for the zebrafish tuft cells. For that reason, we have re-worked the entire manuscript to refer to the *pdia2*-positive subset as Tuft-A cells, and the *alox5a*-positive subset as Tuft-B cells. As both reviewers point out, our findings raise several interesting questions that we hope to pursue in follow-

up work, and it is important that we start those experiments with a clear naming convention. We would like to thank reviewer four for raising this important point.

4. Couple of typos that I happened to notice:
a. Line 305: give -> given
b. Fig 3C, y-axis legend: Bodly > Body
c. Fig 1D, legend still says "Avg. Exp"
We have fixed these errors.

Response to author's point-by-point:
1. The added discussion of mammalian results suggesting that two tuft cells are actually different maturation states of a single cell population are well done.
We appreciated the initial suggestion from the reviewer. This discussion clearly added a new dimension to our findings.

2. The analysis to compare zebrafish tuft cell types is really nicely done, and I think probably extremely informative. Given that this study is establishing the existence of these two zebrafish cell types, I think it's really valuable to identify how they relate to tuft cell types in other animals. For the authors to consider:
a. Given that your results suggest that the 2 tuft-cell populations that you're studying in zebrafish may not be equivalents to the 2 tuft-cell populations or maturation states that exist in mammals, is it worth changing their naming from "tuft 1" and "tuft 2" in your manuscript to something else? "tuft A" and "tuft B" or "tuft pdia2+" and "tuft alox5+"? Since I expect you will be working on these populations for some time, it might help prevent a common type of confusion that you would have to work against – the automatic assumption that your ZF Tuft-1 is the same as the mouse tuft-1. (I do not think the two zebrafish tuft cell populations are any less interesting if they do not perfectly correspond to two mammalian types.)
b. Lines 69 – 75: The use of "like mammals" in these sentences (especially for "resolved into two distinct subtypes" suggests to me here that you're going to make the argument that these cell subtypes are equivalent across animals, which might not be the case? Depending on how you'd like to present this, your readers might find the manuscript clearer
We agree with reviewer four on these points. The comparative work was challenging, but we feel the findings make our work more interesting to a large body of researchers. We also feel the nomenclature suggestion is a particularly important one, particularly considering the data in Figure 2. In the end, we opted to change all text and figures to refer to *pdia2*+ tuft cells as Tuft-A cells, and *alox5a*+ tuft cells as Tuft-B cells. Upon re-reading the manuscript, we have removed "like mammals" from line 69. We feel this is most consistent with the findings from figure 2. However, we have kept "like mammals" in line 75, as we feel the similarities in terms of leukotriene synthesis merit highlighting. Separately, we would like to express our gratitude to reviewer four for their contributions to this aspect of the manuscript. All authors agree that we have benefitted considerably from their input.

3. I think these modifications more accurately describe your study's impressive phenotypes.
We are grateful to the reviewer for their assessment of the phenotypes described in the study. Obviously, there is a lot to work on in the future, but we feel our findings will be of broad interest, and we appreciate the reviewer's support.

4. I am fine with this.
We feel that retaining data on F-actin rich cells, while adding the adult *pou2f3*+ cell counts requested by reviewer two improved the quantitative aspects of Figure 3 and we appreciate the suggestions from both reviewers on this point.

5. I think the changes made to results & discussion with regards to this point have helped clarify the limitations of the approach (which does not decrease the value of the results whatsoever).
All reviewers have made important contributions to our discussion of the limitations of studying systemic loss of RANK function. We agree that it is important to clarify those points, and we look forward to digging into this question more in the future.

6. I am fine with the authors' decision to keep this analysis in their manuscript, and it does provide valuable information. The addition of the results from other groups is also warranted and helpful. However, I think the language is still somewhat mixed up about developmental relationships and transcriptional similarity, which aren't equivalent. The analysis here provides evidence that BEST4 cells might play a role more akin to other secretory cell types, since their transcriptional profile is more similar to other secretory cell types, but doesn't provide evidence that BEST4 cells have secretory origins (though the added references suggest that they do). I think the use of the term "relationship" in this paragraph is vague and confuses the two points, and I think it would be better revised to reflect the analysis done. The authors should feel free to re-write as they wish, but here is a suggested revision that I think more accurately describes your analysis:
a. 307: "their relationship to other intestinal cell types remains unclear, with more recent work" -> ", more recent work"
b. 311-312: "providing a quantitative measure of their relatedness" -> "providing a quantitative measure of their transcriptional similarity"
c. 315: "Our positioning of BEST4 cells within the secretory branch matches the observation" -> "Our positioning of BEST4 cells within the secretory branch shows these cells have a more similar transcriptional profile, and suggests that BEST4 cells may occupy a similar supportive or immune-related functional role in the intestine. This aligns with recent observations that"
This is an important suggestion. In our original drafts, we relied on the word "relatedness" without due consideration of potential meanings. Reviewer four is correct. Our wording could be interpreted to suggest that we are arguing that our data indicate that BEST4 cells have a "secretory" origin. We feel that developmental questions have been more effectively dealt with by the Farrell group (PMID: 41446049) and that our analyses complement those studies by showing transcriptional similarities. To ensure we do not overstate our findings, we have taken all suggestions from reviewer four on board. We believe this approach, along with our citation of PMID: 41446049 is a more effective appraisal of how best to consider developmental and functional relationships between BEST4 cells and all other intestinal epithelial cells.

7. This analysis is very cool and much more supportive of your points than the original one.
We are grateful to reviewer four for their enthusiasm on this point. We feel the comparative work in the final version of the manuscript is considerably better than the initial GO term analyses. After internal discussions, we have agreed to make this approach routine in our lab examination of transcriptomic data, and we appreciate the initial prompt from reviewer four.

8. The more complete methods section is appreciated (and important!).
Thanks!

REVIEWERS' COMMENTS

Reviewer #2 (Remarks to the Author):
The authors have mostly addressed our concerns, which is appreciated. However, two points (raised in the previous round) remain.
We would like to take this opportunity to express our gratitude to reviewer two. We appreciate their ongoing commitment to ensuring our manuscript receives a timely review, while also stimulating important discussions around the implications of our findings.
Reviewer two raises two final points that we highlight below in bold. In both instances, we agree with reviewer two and have made the suggested modifications to the text. We appreciate reviewer two's emphasis of these points and concur that they add important context to the interpretation of our data.

Response to the authors' point-by-point:
#1 (point-by-point #2 in the previous version)
I appreciate that the authors have included a discussion of the potential contributions of immune-derived signals and leukocyte–epithelium interactions as a limitation, which is helpful. It would further improve clarity to note that the observed epithelial phenotypes could, at least in part, be influenced by the inflammatory environment, and that a direct causal role of RANK signaling in epithelial lineage specification therefore remains to be fully established.
We share reviewer two's interest in clarifying this point. To address this concern, we have inserted two additional statements in the manuscript.
1. In the results section, we conclude Figure 4 by stating "Given the impact of intestinal leukocytes on epithelial cell dynamics, we consider it possible that RANK deficiency may, at least partially, affect intestinal epithelial cell specification by altering the inflammatory environment of the gut."
2. In the Limitations section, we now explicitly state "However, our work does not exclude the possibility that RANK may modify the specification of epithelial lineages by altering the inflammatory state of the intestine."
We feel these two statements ensure that our work highlights the possibility of contributions from immune cells, and appreciate the reviewer's contributions to this aspect of our manuscript.

#2 (point-by-point #3 in the previous version)
The authors provide compelling evidence that cftr expression is highly enriched in BEST4 cells under homeostatic conditions, and the cited organoid studies further support a key functional role for CFTR in BEST4 cell–mediated responses to enteric pathogens. These data suggest that BEST4 cells represent a major population mediating CFTR-dependent fluid efflux.
However, it remains unclear whether cftr retains full specificity as a marker of BEST4 cells under infection conditions. While CFTR-dependent responses appear to be functionally restricted to BEST4 cells, this does not exclude the possibility that cftr expression may be modulated in other epithelial populations upon infection. Therefore, an increase in cftr-positive cells following Vc exposure may reflect either expansion of BEST4 cells or changes in cftr expression.
Given that the conclusion of increased BEST4 cells upon Vc infection is relatively novel, careful validation and interpretation are important. In this regard, confirming BEST4 cell identity using best4 expression would further strengthen the evidence for BEST4 cell expansion, as shown by the best4 probe in Figures 6I and 8G and in the cited organoid studies (PMID: 40010349). The authors now state that Vc exposure increases the number of cftr-expressing epithelial cells, which is helpful. However, this statement is followed by the interpretation that this reflects an expansion of BEST4 cells. Given that cftr may not be a fully specific marker under infection conditions, this interpretation could be somewhat overstated.
It would improve clarity to more clearly separate the observation (increase in cftr-positive cells) from the inference (BEST4 cell expansion), or to slightly temper the wording in both the abstract and the main text to avoid overinterpretation.
Addressing or clarifying this point would improve the precision of the conclusions.
We agree with reviewer two that the wording on the point would benefit from slight tempering. To address this point, we modified the manuscript in two places.

1. Our abstract now reads "Functionally, we demonstrate that the number of cells expressing the BEST4 cell marker *cftr* expand following infection with a pandemic strain of *Vibrio cholerae*".
2. The relevant results section now states: "In these experiments, we discovered that exposure to *Vc* led to a significant increase in the number of *cftr*-positive intestinal epithelial cells at both time points (Figure 7D)." As suggested, we have removed the inference that this result reflects an expansion of BEST4 cells. We agree with the reviewer that tempering the wording in this way ensures that our interpretation is not overstated.

Reviewer #3 (Remarks to the Author):
I co-reviewed this manuscript with one of the reviewers who provided the listed reports. This is part of the Nature Communications initiative to facilitate training in peer review and to provide appropriate recognition for Early Career Researchers who co-review manuscripts.
We appreciate reviewer three's contributions to the review of this manuscript.

Reviewer #4 (Remarks to the Author):
The authors have satisfied my (few) remaining comments. I also agree with their responses to the other reviewers. While several of the proposed experiments are excellent lines of inquiry, to me they seem to be the beginning of entirely new studies, rather than being necessary for this already valuable study. The authors explicitly point out the limitations of their genetic tools and the need for additional future work to further address these questions; I think we should accept these already very informative data as-is, and hopefully additional studies will come out in the future that address some of the remaining questions.
We would like to express our gratitude to reviewer four for their contributions to the review process. We share the reviewer's enthusiasm for the suggested follow-up work, and look forward to tackling these studies in the future.
